# Stimuli-responsive nanocarriers for drug delivery, tumor imaging, therapy and theranostics

**DOI:** 10.7150/thno.38069

**Published:** 2020-03-15

**Authors:** Peng Mi

**Affiliations:** Department of Radiology, Center for Medical Imaging, and State Key Laboratory of Biotherapy and Cancer Center, West China Hospital, Sichuan University, No.17 South Renmin Road, Chengdu, 610041, China

**Keywords:** nanoparticles, stimuli-responsive, tumor microenvironment, diagnosis, theranostics, clinical translation

## Abstract

In recent years, much progress has been motivated in stimuli-responsive nanocarriers, which could response to the intrinsic physicochemical and pathological factors in diseased regions to increase the specificity of drug delivery. Currently, numerous nanocarriers have been engineered with physicochemical changes in responding to external stimuli, such as ultrasound, thermal, light and magnetic field, as well as internal stimuli, including pH, redox potential, hypoxia and enzyme, *etc*. Nanocarriers could respond to stimuli in tumor microenvironments or inside cancer cells for on-demanded drug delivery and accumulation, controlled drug release, activation of bioactive compounds, probes and targeting ligands, as well as size, charge and conformation conversion, *etc*., leading to sensing and signaling, overcoming multidrug resistance, accurate diagnosis and precision therapy. This review has summarized the general strategies of developing stimuli-responsive nanocarriers and recent advances, presented their applications in drug delivery, tumor imaging, therapy and theranostics, illustrated the progress of clinical translation and made prospects.

## Introduction

Since the discovery of the enhanced permeability and retention (EPR) effect and impaired lymphatic drainage of tumors 1, nanocarriers have been regarded as promising drug delivery vehicles to tumors 2-5. In general, nanocarriers in the range of 10 to 200 nm are more likely to be accumulated in solid tumors by passively extravasation from the hyperpermeable tumor blood vasculature 6 and the dynamic openings 7. Nanocarriers provide a versatile platform for loading a wide range of payloads, including imaging agents, nucleic acids, anticancer drugs, photosensitizers and antibodies, *etc*., to improve the diagnostic and therapeutic outcomes 8,9. By incorporating bioactive compounds inside nanocarriers, it could avoid enzymatic degradation and undesired exposure to healthy organs, maintain drug activities, as well as alert the half-life in blood circulation, tumor accumulation and biological performance. Until now, several types of nanocarriers have been engineered for drug delivery in oncology 10, 11, including dendrimers, metal nanoparticles (*e.g.*, iron oxide nanoparticles), polymeric micelles, liposomes, inorganic nanoparticles (*e.g.*, silicon nanoparticles), and cell membrane-based nanoparticles *etc*. Currently, some nanocarriers have been approved for cancer treatment in clinic, for instance, the doxorubicin-incorporated PEGylated liposome (*i.e.*, Doxil^®^) is approved for handling Kaposi's sarcoma and ovarian cancer.

Nanocarriers are supposed to deliver bioactive compounds (*e.g.*, imaging or therapeutic agents) to tumor tissues or cancer cells for achieving improved diagnostic and therapeutic efficacy. However, it meets several barriers during circulation or in tumors 12, such as protein corona, degradation, burst release or leaking of cargos, and recognition and clearance by the reticuloendothelial system (RES) *etc*. Several strategies have been applied to address this, including applying PEG shell for achieving stealth effect 13, decorating with targeting moieties or charge conversion materials for improved cellular internalization 14, multistage drug delivery 15, introducing hydrophobic units or cross-link the core to increase the stability, adding specific molecules to escape from RES, *etc*. Although the PEGylated nanocarriers exhibited advantages in prolonged circulation, improved drug solubility and reduced side effects, the delivery efficacy of most nanocarriers is still quite low, which requires further improvement 16. Therefore, strategies for tumor-specific drug delivery have been exploited, mainly including stimuli-responsive nanocarriers 17, and ligand- installed nanocarriers 2, while both were developed to improve the precision of drug delivery but with different focus. The stimuli-responsive nanocarriers are mainly functionalized to delivery, release and activate cargos in specific regions (*e.g.*, tumor microenvironments or intracellular spaces of cancer cells) by responding to internal/external stimuli, *e.g.*, pH, enzymes, *etc*. 18, 19, while the ligand-installed nanocarriers are mainly applied to promote the specific internalization between nanocarriers and specific cells, *e.g.*, cancer cells, tumor vascular endothelial cells 2, *etc*. The stimuli-responsive nanocarriers could specifically delivery cargos into tumor microenvironment or cancer cells, while the ligand-installed nanocarriers could specifically target cancer cells that highly expressing receptors. From the application view, the stimuli-responsive nanocarriers have attracted broad attention, as the stimuli could be existed/generated in most of the tumors, while the cancer cell-specific receptors were reported to be expressed only on partial cancer cells (*e.g.*, the expression of Her2/neu was only found in less than 25% of breast cancer patients) 20, which may require preselection of receptors for the application of ligand-installed nanocarriers. It is possible to develop nanocarriers with stimuli-responsive functions for controlled drug release, and with ligands on their surface for targeting cancer cell. In addition, nanocarriers have also be functionalized for cancer theranostics, as the combination of diagnostics and therapy was generally referred as “theranostics” 16, 21, which could be achieved by loading both diagnostic and therapeutic compounds inside the same nanocarriers 22.

**Figure 1:**
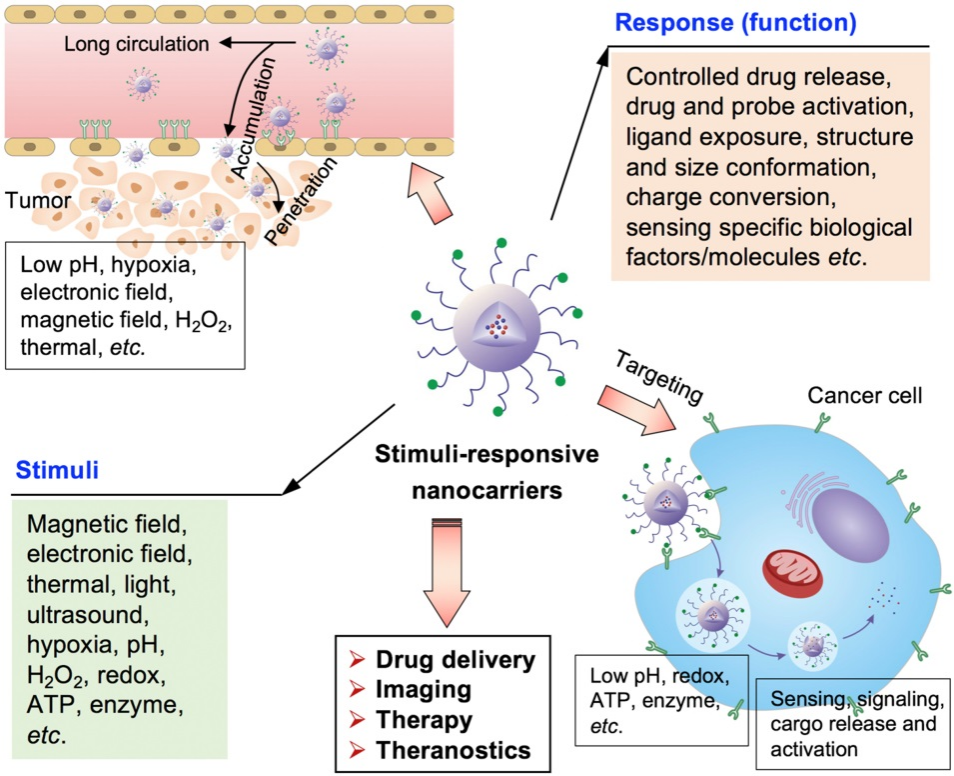
** The stimuli-responsive nanocarriers for drug delivery to tumors towards precision imaging, effective therapy and theranostics.** The nanocarriers could accumulate and penetrate tumors, and target cancer cells for achieving different applications and functions by responding to the external and internal stimuli.

The stimuli-responsive nanocarriers have been rationally designed and developed by considering different pathological profiles in normal tissues, intracellular compartment and tumor microenvironment, to increase drug delivery specificity, efficacy and biological activities (Figure 1) 23-29. In general, the nanocarriers could response to external stimuli, including magnetic field, temperature (*i.e.*, thermal), ultrasound, light (*e.g.*, laser) and electronic field, *etc.*, and internal stimuli, including pH, ATP, H_2_O_2_, enzyme, redox-potential, and hypoxia *etc*., while the stimuli could be appeared in tumor microenvironment or inside cancer cells (Figure 1). The stimuli- sensitive functions facilitate on-demand or controlled drug release, promoted tumor accumulation, ligand exposure, drug or probe activation, nanoparticle structure or size conformation, charge conversion, as well as signaling in specific positions, sensing of special pathological factors/molecules, tumor-specific diagnosis and theranostics (Figure 1). Moreover, the external force (*i.e.*, stimuli) could also alert the biological performances of nanocarriers, for example, the external magnetic field could increase the accumulation of magnetic nanocarriers in tumors. Furthermore, the stimuli could also be applied to provoke biological activities of certain prodrug- formulated nanocarriers in diseased regions/cells for precision therapy. In addition, the stimuli-responsive nanocarriers were reported to overcome multidrug resistance in cancer treatment 30.

This review has summarized recent progress and achievements in nanocarriers that responsive to external or internal stimuli, presented different stimuli-sensitive strategies and their applications in drug delivery, tumor imaging, therapy and theranostics. In the following sections, the clinical translation of stimuli-responsive nanocarriers has been illustrated, and finally the perspectives were made.

## External-responsive nanocarriers

The external stimuli, mainly including thermal, magnetic field, electronic field, ultrasound and light, could affect the fate of nanocarriers inside the biological systems. With the external stimuli, it facilitates enhancing the accumulation of nanocarriers in desired regions with outer forces (*e.g.*, magnetic field), controlled release, intracellular drug delivery, as well as activated imaging and therapy. There are several advantages of applying external-stimuli for drug delivery to tumors: (1) it could precisely control the location and intensity of given external stimuli (*e.g.*, magnetic field, laser irradiation); (2) the external stimuli could be added or removed depending on the treatment requirement; (3) multiple external stimuli could be overlaid for achieving multifunction in cancer theranostics; (4) the possibility to provide multi-times or continuous (*e.g.*, several hours or days) stimuli for drug delivery and therapy. However, the externally-directed triggers would be impractical for accessing and treating the metastatic lesions, when their location is uncertain. Here, the application of external stimuli-responsive nanocarriers will be discussed in this section.

### Ultrasound-responsive nanocarriers

Ultrasound is a type of high-frequency sound waves, which could affect nanocarriers for controlled drug release at diseased sites (*i.e.*, tumors). The intensity of ultrasound could be adjusted for different applications. At low ultrasound frequencies (< 20 kHz), it could be applied for imaging, while it could be applied for disrupting nanocarriers to release cargos or enhancing the permeability of cancer cell membrane at high ultrasound frequencies (> 20 kHz) 31. Until now, several microbubbles have been commercialized, such as Albunex, Optison, Definity, Imagent, Levovist and Sonazoid *etc* 32. However, the large size (1-10 μm), short half-life and low stability of microbubbles limit their access to vascular compartments in tumor tissues and deep penetration. Several size switchable microbubbles (*i.e.*, from microbubbles to nanobubbles) 33, or nanocarriers have been engineered for ultrasound imaging 34, ultrasound- triggered drug delivery 35-37, and ultrasound- triggered cancer theranostics (Table 1), including nanobubbles 38, calcium carbonate (CaCO_3_) nanoparticles 39, liposome 40, nanodroplets 41, vesicles 42 and nanoparticles 43, *etc*. Generally, the ultrasound-sensitive nanocarriers are incorporating gas or contrast agents 44, including air, N_2_ and perfluorocarbons, *etc*., or generating gas in biological environment 45-47, such as CaCO_3_ nanoparticles 39.

**Figure 2:**
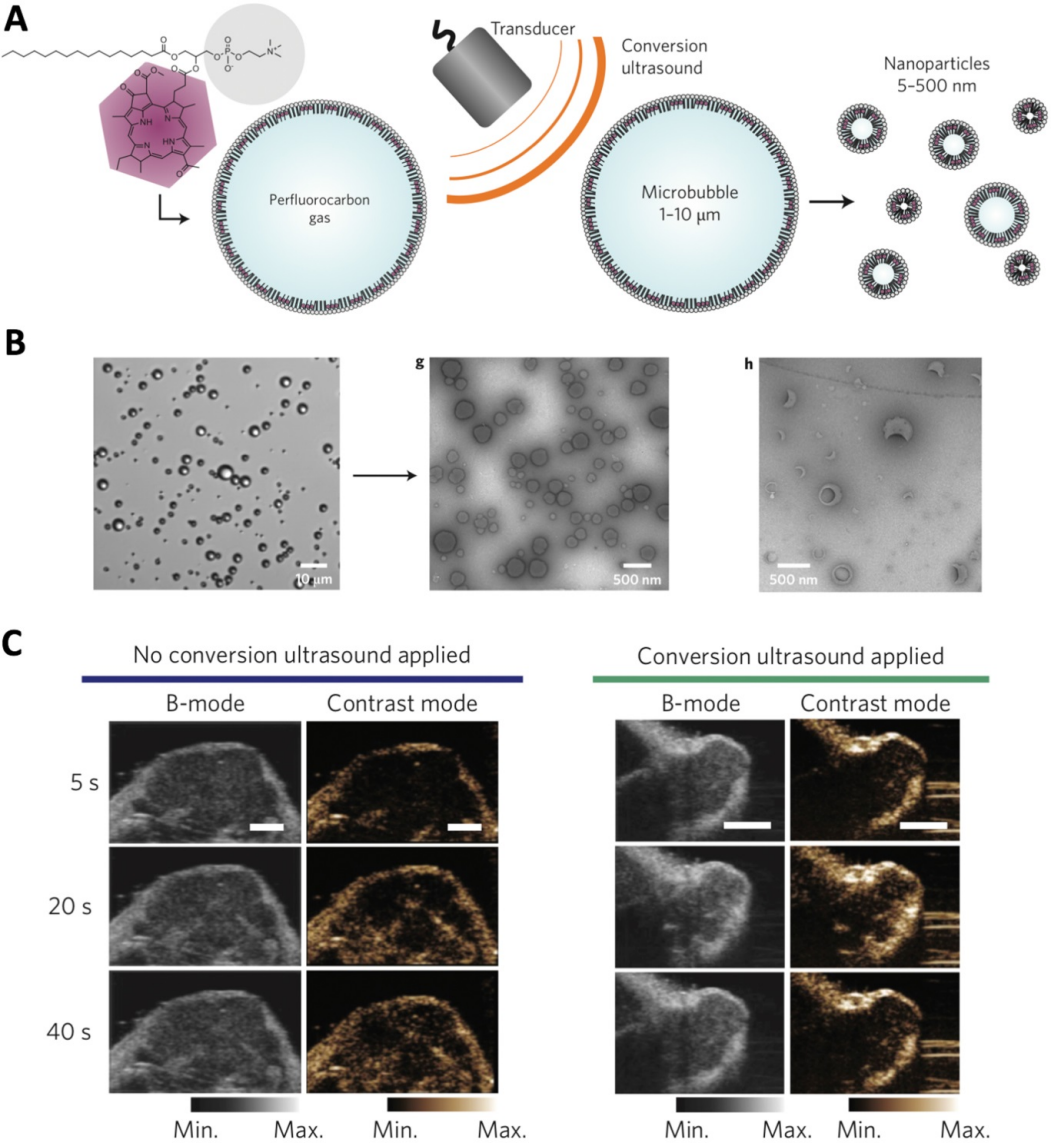
** The ultrasound-triggered conversion of microbubbles to nanoparticles for multimodality tumor imaging. (A)** Illustration of ultrasound-triggered conversion of porphyrin microbubbles to nanobubbles. **(B)** Confirmation of the conversion of microbubbles to nanobubbles with ultrasound stimuli by microscopy. **(C)** Ultrasound imaging of tumors by using no conversion ultrasound (left) and by administration of conversion nanoparticles (right). Adapted with permission from ref. 33, copyright 2015 Springer Nature Publishing AG.

The ultrasound-responsive nanocarriers could be applied for tumor ultrasound imaging, which is safe, low cost and widely applied in clinic, and providing images with high spatial resolution. The gas and contrast agent (*e.g.*, perfluoropentane) incorporated nanocarriers 48, as well as nanoparticles that could generate gas (*e.g.*, CO_2_) in biological environment 34, 49, have demonstrated tumor-specific imaging at high resolution and intensity. In another strategy, the porphyrin microbubbles (1-10 μm) could be converted into nanobubbles (5-500 nm) for tumor ultrasound imaging (Figure 2) 33. Besides, ultrasound could also be applied for triggering controlled release of cargos (*e.g.*, imaging probes, anticancer agents) from nanocarriers at desired tumor sites 42, 50. For example, the phase changeable, polymeric nanodroplets could be generated for tumor imaging and doxorubicin release due the collapse of microbubbles when responding to the low-intensity focused ultrasound 41. Moreover, the ultrasound-responsive property could be applied for enhancing the tumor accumulation and intracellular delivery of bioactive compounds (*e.g.*, siRNA, DNA) 51. Because ultrasound could increase gap in tumor vasculature wall (*i.e.*, disrupting of vascular integrity) to facilitate extravasation of drug delivery systems to malignant tissues, as well as enhance cellular uptake by cancer cells 52-54. However, the large size of ultrasound-sensitive nanocarriers may limit their penetration across tumor tissues, due to the weak penetration of large nanocarriers 6. In addition, the drug- loaded, ultrasound-sensitive nanocarriers could further be applied for cancer therapy 55, imaging- guided therapy 56-58, and theranostics 39, 59.

### Thermal-responsive nanocarriers

**Figure 3:**
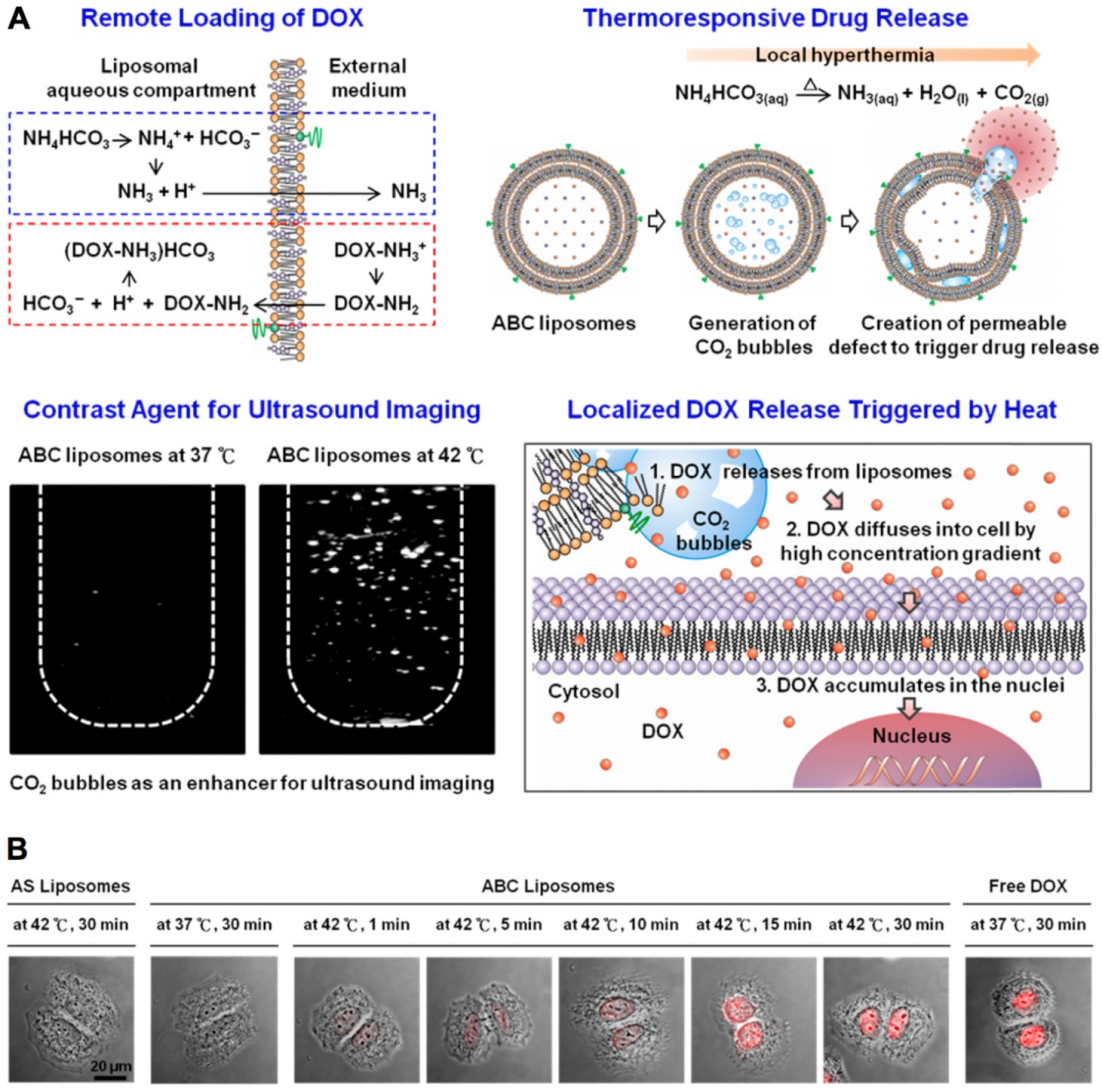
** Thermal-sensitive nanocarriers for drug delivery. (A)** Thermal-sensitive liposomes (*i.e.*, ABC liposomes) for molecular imaging, drug delivery and controlled drug release. **(B)** Cellular uptake of thermal-sensitive liposomes, control liposomes (*i.e.*, AS liposomes) and free doxorubicin. Adapted with permission from ref. 64, copyright 2013 American Chemical Society.

The temperature-sensitive nanocarriers have also been widely applied for drug delivery and dealing with cancer. Generally, the nanocarriers are designed to be stable in normal regions with temperature up to 37 °C and sensitive to higher temperature (> 40 °C) with significantly changes in their properties by responding to the narrow temperature shift. Until now, several thermal-sensitive nanocarriers have been formulated (Table 2), including liposomes 63-65, polymeric micelles 66-70, nanocomposites 66, 71, nanocapsules 72, nanogels 73-76 and vesicles 77, 78, *etc*. The thermal-sensitive nanocarriers is generated with materials that could undergo physicochemical properties variation associating with temperature change 71, 79. The temperature- sensitive materials are mainly including poly(N- isopropylacrylamide) (PNIPAM) 80, 81, poly(N-inyl isobutyramide) (PAMAM) 82, poly(2-oxazoline) (POxs) 83, and poly [2-(2-methoxyethoxy) ethyl methacrylate] [PMEOMA] 84, *etc*. Besides, another strategy for achieving thermal-sensitivity is to incorporate thermal-unstable materials inside nanocarriers. For instance, the NH_4_HCO_3_ incorporated liposome could generate CO_2_ after giving local hyperemia (42°C) to make liposome swollen and collapse 64, leading to drug release for efficient intracellular drug delivery (Figure 3).

The thermal-sensitivity nanocarriers could be applied for gene and drug delivery by using thermal- sensitive polymeric materials 63, 85, 86, which could shift from hydrophilic to hydrophobic for forming nanocarriers. In a recent study, the siRNA-SS- PNIPAM conjugates could form siRNAsomes by self- assembly at higher temperature (> 32°C) than the lower critical solution temperature (LCST) for phase transition 78. In another study, the nanocarriers with PNIPAM on the surface formed micellar networks (*i.e*., aggregates) at temperature higher than LCST, while disassociated to each other at low temperature 75. In this way, the thermal-sensitive nanocarriers could also be applied for plasmid DNA (pDNA) condensation 84, folding proteins 77, and incorporating hydrophobic anticancer drugs (*e.g.*, doxorubicin) 66. Besides, it could be applied for controlled releasing cargos in diseased regions with local hyperemia 64, 67, 85. For instance, the doxorubicin could be released from the lipid-peptide vesicle by responding to mild hyperemia 87, as the peptides in the wall of vesicles could open pores at high temperature (42.5°C). In another case, the Nile Red and doxorubicin could be release from the polymeric micelles by responding to the thermal-stimuli, where the poly(γ-2-(2-(2-methoxyethoxy)ethoxy)ethoxy-ε- caprolactone)-*b*-poly(γ-octyloxy-ε-caprolactone) (PMEEECL-*b*-POCTCL) diblock copolymer displayed phase transition at temperature above its LCST (38 °C) 67. The thermal-sensitive polymeric micelles displayed higher cellular uptake at high temperature (42.5°C) than at normal temperature (37 °C), as well as lower survival than free doxorubicin as tested on MCF-7 cancer cells. Although with much advances in developing temperature-sensitive nanocarriers, only limited thermal-sensitive materials are existed, which requires further development. The thermal-sensitive temperature of some materials and nanocarriers was neither in the range of biological systems (*e.g.*, 37- 42°C), nor could be simply shifted to another desired temperature. It further has to point out that some thermal-responsive nanocarriers were developed with non-biodegradable polymers (*e.g.*, PNIPAM), which may be difficult for clinical translation. Thus, development of biodegradable and thermal-sensitive materials would be a future direction. In addition, the accumulation of nanocarriers in tumors is still critically important for achieving pinpoint thermal- triggered drug release and therapy.

### Magnetic-responsive nanocarriers

The magnetic-responsive nanocarriers have been engineered, as the magnetic nanoparticles has intrinsic tropism to magnetic field for tumor targeting, while it also could generate local hyperthermia under an alternating magnetic field for triggering drug release and tumor ablation. Until now, several magnetic-responsive nanocarriers have been formulated (Table 3), including magnetic nanoparticles 89, 90, liposomes 91, superparamagnetic iron-oxide nanoparticles (SPIONs) 92, polymeric micelles 93, albumin nanocapsules 94, magnetic nanocarriers 95, 96 and magnetic nanogels 97, *etc*. Generally, nanocarriers are incorporating magnetic materials for achieving magnetic-sensitivity, which are mainly including iron oxide nanoparticles (*e.g.*, Fe_3_O_4_ nanoparticles) 98, iron oxide hybrid nanoparticles (*e.g.*, graphene/Au/Fe_3_O_4_ hybrids) 99, and other magnetic nanomaterials (*e.g.*, ZnFe_2_O_4_) 100. The incorporated magnetic materials also could be applied for tumor imaging by magnetic resonance imaging (MRI) 92, 101, 102. Besides magnetic materials, the contrast agents 103, anticancer drugs 101, 104, plasmids 100, antibodies 98 and photosensitizer 91, could also be incorporated inside the magnetic-sensitive nanocarriers for achieving multiple functions or multimodal therapeutic effects. Moreover, the nanocarriers could be engineered for passive tumor targeting through the EPR effect 105, as well as be installed with targeting moieties (*e.g.*, folic acid) for active targeting cancer cells 94.

**Figure 4:**
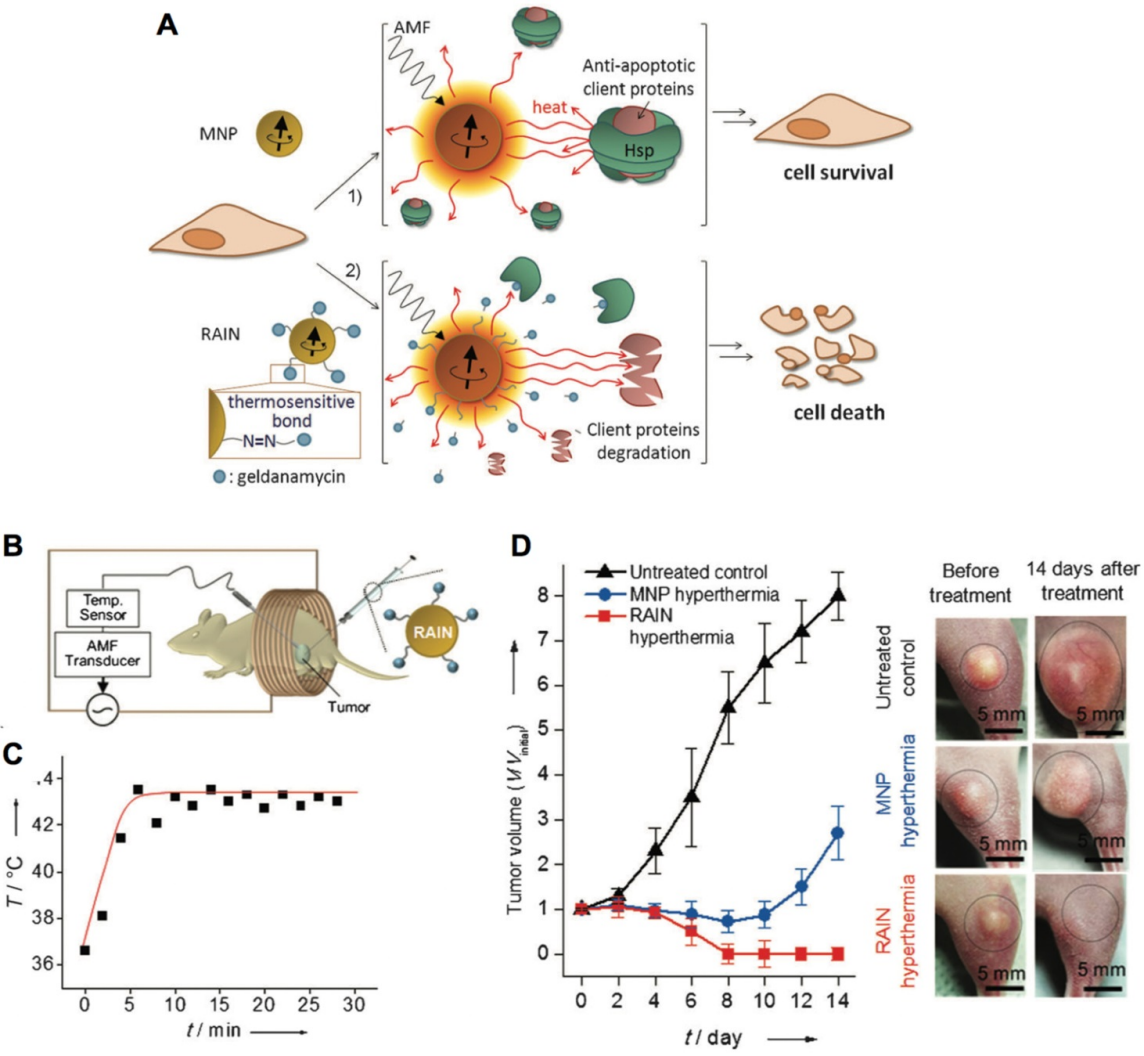
** Magnetic-responsive nanocarriers for tumor therapy. (A)** Schematic illustration of resistance-free apoptosis-inducing magnetic nanoparticles (RAIN) for cargo release and killing cancer cells. **(B)** Illustration of applying magnetic-sensitive nanocarriers for tumor treatment in an alternating magnetic field. **(C)** The temperature profiles in tumors. **(D)** The anti-tumor efficacy by magnetic-sensitive nanocarriers with hyperthermia. Adapted with permission from ref. 89, copyright 2013 WILEY-VCH Verlag GmbH & Co. KGaA, Weinheim.

**Figure 5:**
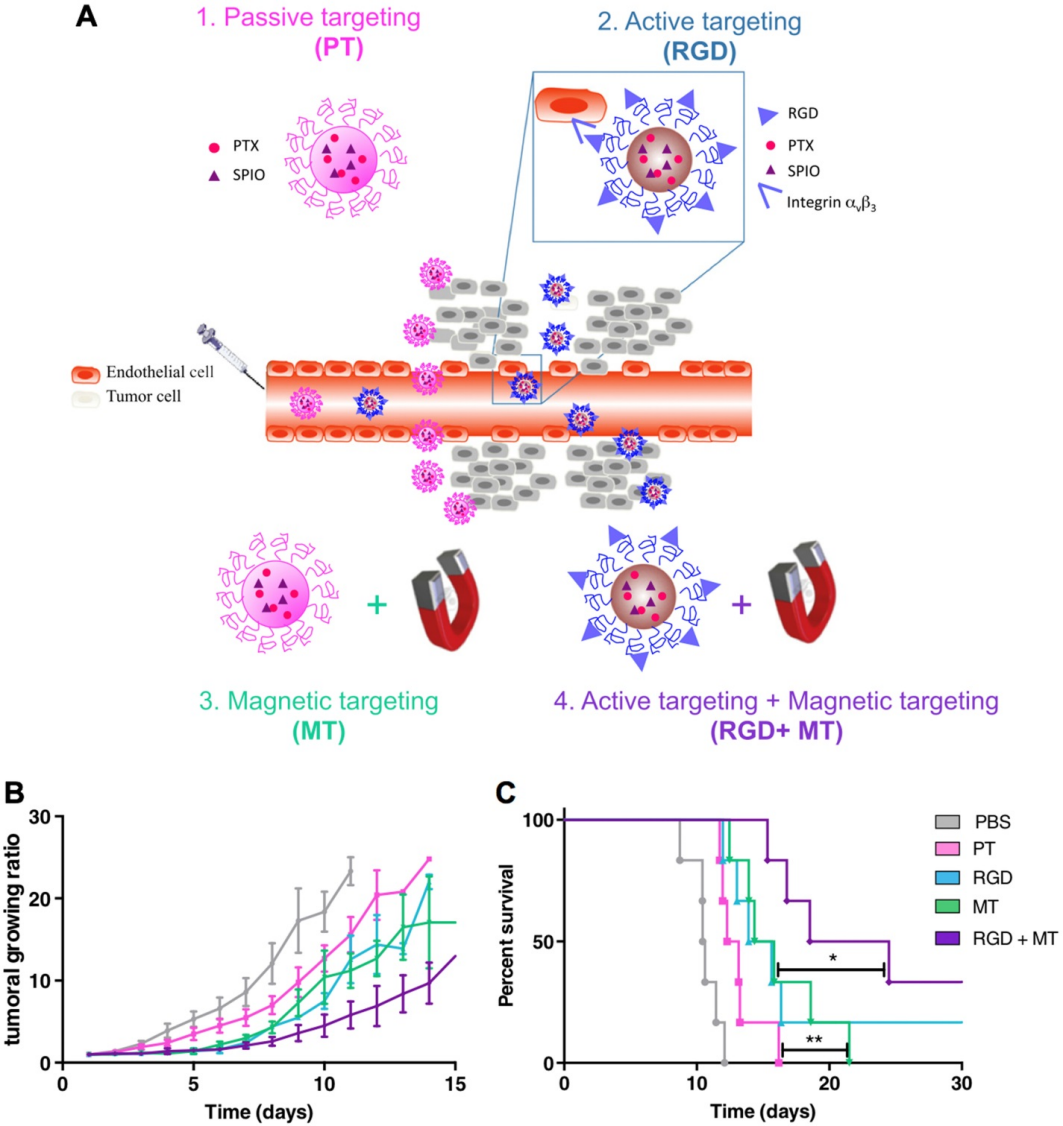
** Nanocarriers for magnetic targeted tumor therapy. (A)** Illustration of paclitaxel (PTX) and SPIO-loaded nanocarriers for tumor passive targeting (PT), active targeting of α_v_β_3_ integrins with installed RGD ligands (RGD), magnetic field (1.1 T)-guided tumor targeting (MT), and combination of magnetic targeting and active targeting (RGD+MT). **(B,C)** The tumor growth ratio (B) and survival rates (C) of CT26-tumor bearing mice. Adapted with permission from ref. 114, copyright 2014 Elsevier B.V.

The interaction between magnetic nanocarriers and magnetic field facilitates the magnetic-guided accumulation of nanocarriers in tumors, while a typical approach is to locate a permanent magnetic field in malignant tissues after administration 94. For example, much higher level of SPIONs and doxorubicin-loaded nanocarriers in tumors have been achieved with external magnetic field-guided tumor targeting, leading to effective tumor ablation 95. In this way, it could be applied for promoting the accumulation of a myriad of bioactive compounds in tumors, including genes, anticancer drugs, and imaging probes 106. Besides, the alternating magnetic field-triggered hyperthermia could induce on-demand release of cargos from the magnetic- sensitive nanocarriers in diseased regions (*i.e.*, tumor or cancer cells) 105, 107, 108. Using hyperthermia to cleave the thermosensitive bonds , the magnetic nanoparticles could release the heat shock protein inhibitors (*i.e.*, geldanamycin), which could block the protective function of heat shock proteins to induce resistance-free apoptosis for effective tumor ablation (Figure 4) 89. This magnetic-sensitive nanocarriers would facilitate treating tumors that resistant to hyperthermia therapy, and overcoming multi-drug resistant (MDR) of cancers. Moreover, the hyperthermia generated by magnetic-sensitive nanocarriers could further be applied for tumor ablation 90, 100, as hyperthermia could induce apoptosis of cancer cells. For example, the magnetic-responsive nanocarriers have been developed with ZnFe_2_O_4_ inside the core and decorated with cationic polymers of polyethyleneimine (PEI) to interact with plasmids on the surface 100. It facilitated cellular uptake of plasmids by the adipose-derived mesenchymal stem cells (MD-MSCs), which could migrate to tumors guided by an alternating magnetic field for effective therapy. Besides primary tumors, the magnetic- responsive nanocarriers have also demonstrated high potential for treating metastatic tumors (*e.g.*, lung metastasis) 109. Furthermore, the magnetic-sensitive nanocarriers could be applied for tumor theranostics 110, as it could probe tumors by MRI or other imaging modalities, and remotely and non-invasively eradicate tumors with the generated hyperthermia in the alternating magnetic field 111. For example, the PEGylated MoS_2_/Fe_3_O_4_ nanocomposites (MSIOs) made through a two-step hydrothermal method, have demonstrated high potential for tumor diagnosis by T_2_-weighted MR imaging and photoacoustic tomography (PAT) imaging, and magnetic-targeted effective photothermal ablation of tumors 112. Meanwhile, it further allowed both T_1_- and T_2_-weighted MR imaging of tumors by doping Mn into the core of Fe_3_O_4_@MoS_2_ nanocomposites (*i.e.*, multifunctional nanoflowers) 113. Some other bioactive compounds, such as photosensitizer chlorin e6 (Ce6), could also be incorporated into the magnetic-sensitive nanocarriers for multi-functional cancer theranostics 96. In addition, the superparamagnetic materials in magnetic-responsive nanocarriers could be extensively employed as a target moiety for improved tumor therapy, which is comparable to the decoration of active targeting moieties. As presented in a recent study, the paclitaxel (PTX) and SPIO-loaded poly(lactic-co-glycolic acid) (PLGA) nanocarriers have been engineered for tumor passive targeting by EPR effect, active targeting of α_v_β_3_ integrins on cancer cells with RGD ligands (RGD), magnetic field (*i.e.*, 1.1 T) guided tumor targeting (MT), and the combination of magnetic targeting and active targeting (RGD+MT) (Figure 5A) 114. Accordingly, both RGD and magnetic targeting drastically exhibited much higher tumor accumulation (*i.e.*, 8-fold increase) of nanocarriers than passive targeting, leading to effective tumor ablation and improved survival rates of colon CT26 tumor-bearing mice, while the combination of magnetic targeting and active targeting demonstrated the best performance in tumor ablation than other groups (Figure 5B,C). Notably, higher accumulation in tumors and lower deposition in livers/lungs have been achieved by magnetic field-guided targeting nanocarriers than the RGD-installed nanocarriers, demonstrating the promise of magnetic targeting approach. Overall, the magnetic field guided- targeting strategy requires tumor-specific drug delivery, as it may also affect normal organs/tissues that distributed with magnetic nanocarriers when exposed to the alternating magnetic field. In addition, the generation of hyperthermia requires high level of magnetic-sensitive nanocarriers in diseased regions, which should be located in the alternating magnetic field. This approach may facilitate treating tumors located in partial regions of the body (*e.g.*, legs, feet and arms, *etc*.), due to safety consideration.

### Light-sensitive nanocarriers

Nanocarriers that could responsive to light have also been extensively developed, as light is an attractive stimulus with the possibility to adjust the irradiation wavelength, power and affecting area 117. In general, the light irradiation, such as UV-Vis and near-infrared light (NIR), could remotely affect the light-sensitive nanocarriers in biological systems (*e.g.*, cancer cells, or tumors). Meanwhile, the light- triggered tumor therapy could be precisely conducted by control the range of irradiation to avoid or minimize potential harm to normal organs and tissues. Until now, several light-responsive nanocarriers have been exploited (Table 4), including polyion complex vesicles (PICsomes) 118, polyplexes 119, 120, nanoparticles 121, 122, polymeric micelles 123, 124, upconverting nanoparticles (UCNPs) 125,126, polymersomes 127,128, liposomes 129, 130, nanogels 131, nanorods 132, and nanorattles 48, *etc*. Meanwhile, the cargos/materials with light-response function could be applied for constructing light- sensitive nanocarriers, such as photosensitizers (*e.g.*, IR780) 133, gold nanocomposites (gold nanoparticles) 134, UCNPs 123, organic molecules (*e.g.*, azobenzene) 135, graphene 131, carbon nanotubes 136-138, and two-dimensional (2D) transitional metal nanomaterials (*e.g.*, MoS_2_, WSe_2_ and WS_2_) 139, 140, *etc*. Nanocarriers could response to light for several activities: (1) alert the conformation of certain molecules, such as azobenzene, spiropyran, dithienylethene and diazonaphthoquinone *etc.* 141; (2) cleave the light-sensitive chemical bonds for nanocarriers disassociation 123; (3) trigger release of therapeutics from nanocarriers in diseased regions 130; (4) light-activated imaging (*e.g.*, photoacoustic imaging) or imaging-guided therapy 142-146; (5) generate singlet oxygen (O_2_^1^), also referred as reactive oxygen species (ROS) for photodynamic therapy (PDT) 147, 148, and photothermal effect for tumor ablation by photothermal therapy (PTT) 149, 150.

**Figure 6:**
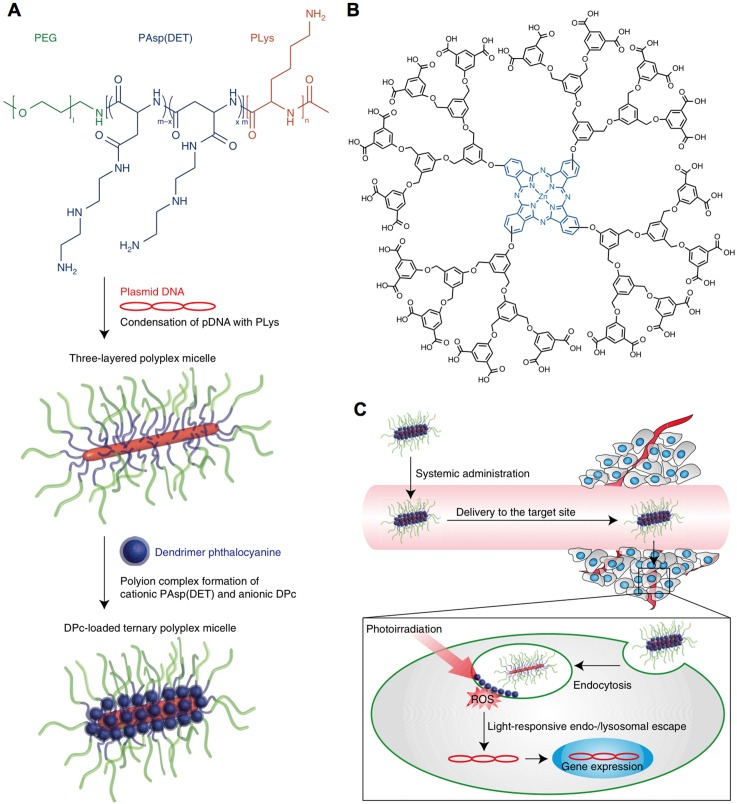
** Schematic illustration of light-responsive nanocarriers for gene transfer. (A)** Preparation of pDNA and photosensitizer-loaded nanocarriers. **(B)** Chemical structure of photosensitizer; **(C)** Light-triggered endo-/lysosomal escape for gene transfection inside cancer cells. Adapted with permission from ref. 119, copyright 2015 Springer Nature Publishing AG.

Nanocarriers could also be formed or assembled by responding to light, due to change the hydrophilic- hydrophobic balance or structure conversion of light-sensitive materials. Recently, the light-sensitive nanoparticles were formed by using 1,2-distearoyl-sn- glycero-3-phosphoethanolamine-*N*-carboxy(polyethylene glycol) (DSPE-PEG) to incorporate spiropyran in visible or dark conditions, and disassociated responding to UV irradiation due to the conversion of SP to merocyanine (MC) 121. The photo-switching nanocarriers demonstrated high potential for loading different bioactive compounds for UV-Vis triggered drug release, including paclitaxel, docetaxel and doxorubicin *etc.*, as well as for cancer therapy 151. The light-switching function also could be applied for inducing reversible aggregation of nanoparticles (*e.g.*, vesicles) 152. However, the short wavelength of UV-Vis may limit their applications. Therefore, the NIR light-sensitive nanocarriers have also been engineered for controlled drug delivery 153, and penetrating into deep tissues 154. For example, the IR-780-incorperated polymeric micelles could response to NIR for doxorubicin release 155. Besides, the light-sensitive nanocarriers facilitate intracellular delivery of bioactive compounds, including genes 120, photosensitizers 118, and anticancer drugs 124, *etc*. In a recent study, the photosensitizer Al(III) phthalocyanine chloride disulfonic acid (AlPcS2a)- incorporated polyion complex vesicles (PICsomes) could sensitive to laser irradiation for endosome escape and drug release, exhibiting much stronger photocytotoxicity than that of AlPcS2a 118. In another strategy, by co-administration of photofrin, it could also induce photochemical internalization (PCI) for achieving endosomal escape of nanocarriers to improve the therapeutic effects of camptothecin 124. Moreover, the light-triggered endosome/lysosome escape also plays an important role in transferring genes into cytoplasm, as genes could be degraded in the late lysosomes to lose activity. For example, the light-responsive, three-layered polyplex micelles have been developed with polycationic polymers to condensate pDNA and load dendrimer phthalocyanine (*i.e.*, photosensitizer), demonstrating efficient systemic gene transfection by light-triggered PCI for endosomal/lysosomal escape (Figure 6) 119.

Furthermore, the light-sensitive nanocarriers could further be activated for imaging-guided tumor therapy 156, 157 and theranostics 60, 156, which could figure out the cut-edge of tumors for precisely irradiation by PTT or PDT. In addition, the light- sensitive nanocarriers could be applied for tumor ablation, as a result of light-triggered generation of ROS and photothermal effect 130, 156, or combined with other bioactive agents (*e.g.*, anticancer drugs) for multimodal cancer theranostics 155, 158. It has also demonstrated high efficacy for treating MDR cancers 134. In general, the light- sensitive nanocarriers have demonstrated high potential for drug delivery, controlled drug release and cancer theranostics, especially tumors that could be accessed by light/laser due to the limitation of light penetration.

## Internal stimuli-responsive nanocarriers

Specific biological factors in tumor microenvironment or inside cancer cells, such as enzymes, ATP, low pH, redox-potential and hypoxia, *etc*., could be specific triggers for controlled drug release, endosome/lysosome escape, prodrug activation, tumor specific imaging and therapy 161. The internal triggers are intrinsically existed in tumor microenvironment or inside cancer cells. However, they usually show poor specificity and heterogenetic distribution in tumors, which may affect the efficacy of internal stimuli-sensitive nanocarriers. In this section, recent advances in nanocarriers responding to internal stimuli, mainly including pH, hypoxia, redox and enzymes, for tumor theranostics will be focused.

### pH-responsive nanocarriers

**Figure 7:**
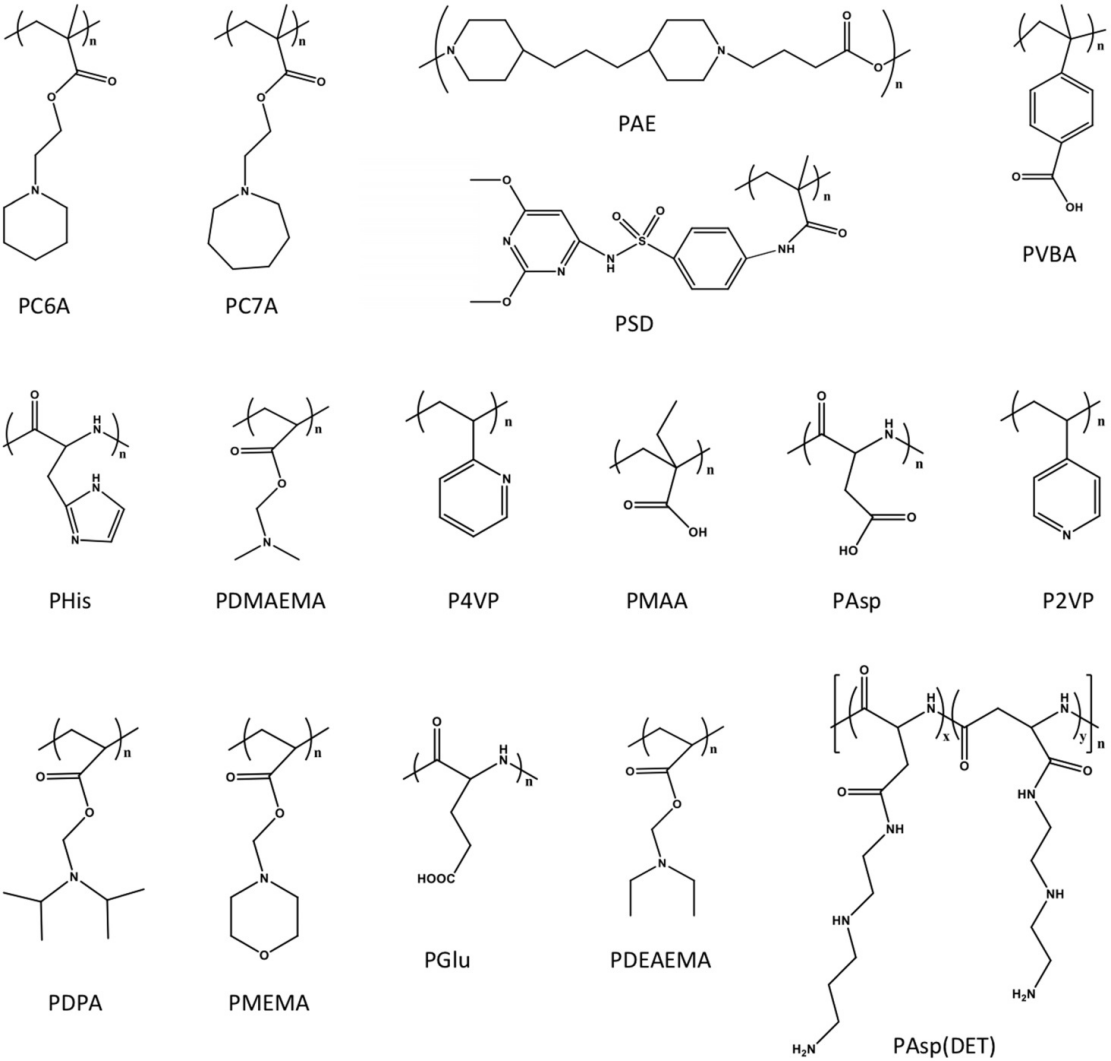
The intracellular or tumor microenvironment pH-responsive polymers have been applied for engineering pH-sensitive nanocarriers.

**Figure 8:**
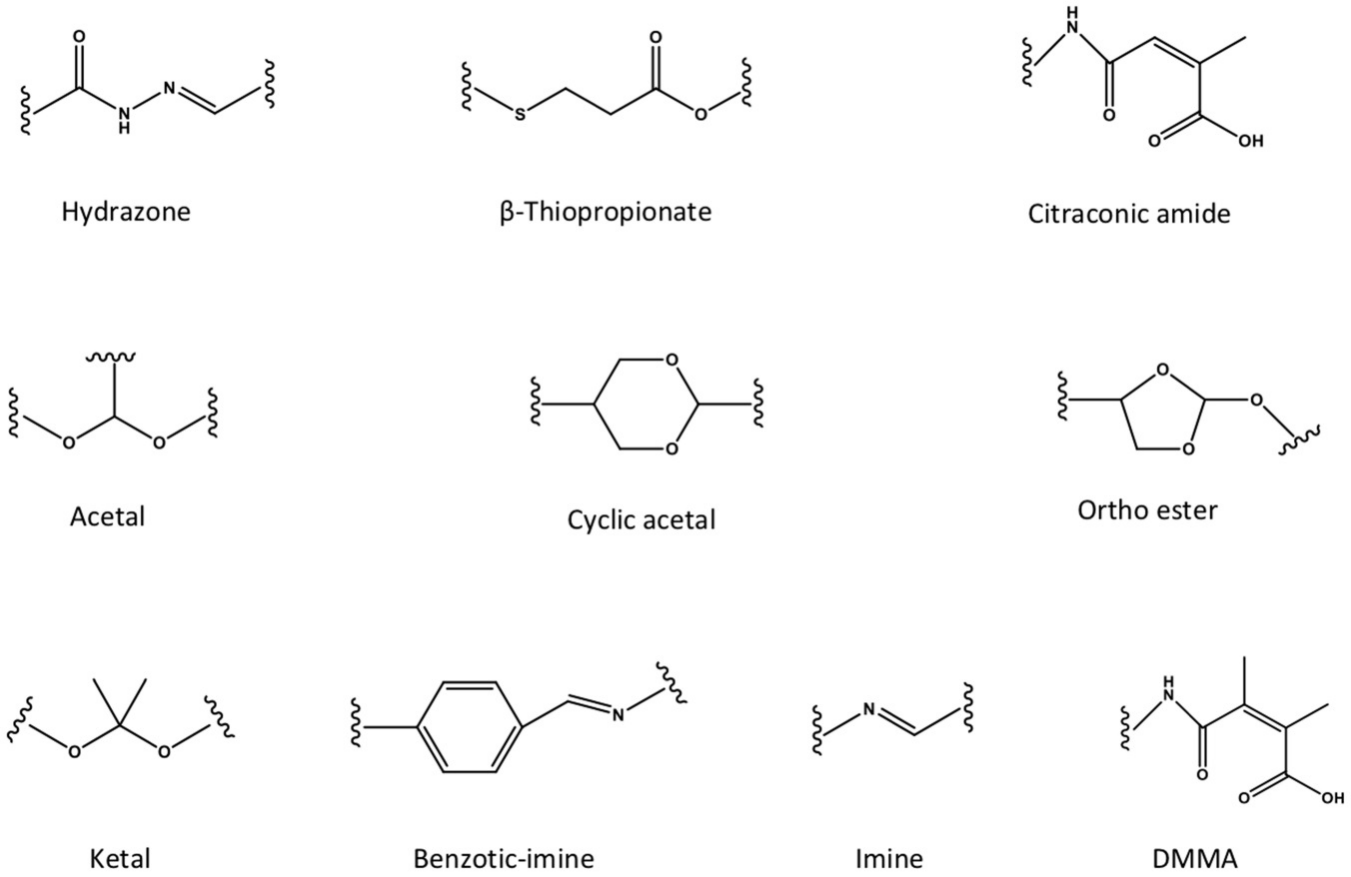
The pH-responsive chemical bonds have been utilized for developing pH-sensitive nanocarriers.

The pH-responsive nanocarriers have been extensively exploited, due to the nature of low pH inside the organelles (*e.g.*, lysosomes and endosomes) of cancer cells and in tumor microenvironment. In general, the pH in cytoplasm, blood and normal tissues is almost around pH 7.0 to 7.4, while it exhibits approximately pH 6 to 4 in endosomal/lysosomal organelles, and pH 6.5 to 6.8 in tumor microenvironment 162. Thus, the pH-responsive in tumor microenvironment could be applied for controlled drug release or prodrug activation, while keep the “stealth effect” of nanocarreirs in normal regions (*e.g.*, in blood circulation) without leaking of cargos. This would decrease the risk of exposure normal organs (*e.g.*, heart) to the toxic cargos (*e.g.*, doxorubicin), and specifically deliver them to tumors for achieving high therapeutic performance. Until now, several types of pH-sensitive nanocarriers, including CaCO_3_ nanoparticles 163, 164, calcium phosphate (CaP) nanocarriers 165-167, inorganic nanoparticles or crystals 168-170, polymer-drug conjugates 171, 172, polymeric micelles 173-175, liposomes 176, polymersomes 177, nanogels 178- 180 and dendrimers 181, *etc*., have been exploited for imaging, intracellular drug delivery, charge conversion, and controlled drug release in tumor- microenvironment 172, 182. Meanwhile, several pH-sensitive polymers have been synthesized for fabricating nanocarriers with pH-responsibility 183, 184, including poly(2- (pentamethyleneimino) ethyl methacrylate) (PC6A), poly(2-(hexamethyleneimino) ethyl methacrylate) (PC7A), poly(β-amino ester) (PAE), poly- sulfadimethoxine (PSD), poly(L-histidine) (PHis), poly(4-vinylbenzoic acid) (PVBA), 2,3-dimethylmaleic anhydride (DMMA), poly(N,N-dimethylaminoethyl methacrylate) (PDMAEMA), poly(N,N-diethylamino- 2-ethylmethacrylate) (PDEAEMA), poly(N'-(N-(2- aminoethyl)-2-aminoethyl) aspartamide) [PAsp (DET)], poly(2-diisopropylaminoethyl methacrylate) (PDPA), poly [(2-N-morpholino) ethyl methacrylate] (PMEMA), poly(4-vinylpyridine) (P4VP), poly (glutamic acid) (PGlu) 185, poly (methacrylic acid) (PMAA), poly(L-aspartic acid) (PAsp) and poly(2- vinylpyridine) (P2VP) (Figure 7). Meanwhile, certain pH-sensitive chemical bonds have also been applied for drug conjugation, confirmation/ size change and charge conversion, *etc*. (Figure 8), which facilitate pH-triggered drug release, and disassociation of nanocarriers inside cancer cells or in tumor microenvironment 186.

Compared to cytoplasm with an almost neutral pH (pH 7.2), the pH in endosomal/lysosomal organelles was around pH 6 to 4. Generally, nanocarriers enter into cancer cells through the pathway of endocytosis, which requires endosome/lysosome escape to avoid further degradation in late lysosomes with low pH. Currently, several intercellular pH-triggered nanocarriers have been engineered for liberating cargos inside cancer cells 187. The pH-triggered charge conversion nanocarriers have also been engineered for intracellular drug delivery, where the neutral or negative charged nanocarriers could turn to be positively charged by responding to low pH in endosomes/lysosomes for disrupting endosomes/lysosomes, due to the protonation of the cationic materials 188, 189. The pH-triggered charge conversion could be obtained with certain chemical groups, such as citraconic anhydride, 2,3-dimethylmaleic anhydride (DA), cis-aconitic anhydride, carboxy dimethylmaleic anhydride (CDM) and cis-4-cyclohexene-1,2-dicarboxinic anhydride, *etc.* The charge conversion strategy facilitates intracellular delivery of antibodies 190, proteins 189, 191, siRNA 192, 193, and DNA 194, as well as enhancing the tumor accumulation of nanocarriers 195, *etc*. As presented in a recent study, the pDNA- loaded nanocarriers (HA-NPs) were innovated by using PAsp(DET) for formulating cationic PAsp (DET)/pDNA condensates and endosome escape, as well as installing hyaluronic acid (HA) for active targeted gene therapy of cancer 196. The HA-NPs could selectively internalize with CD44 receptors overexpressed on B16F10 melanoma cancer cells and tumor vascular endothelial cells to prompt preferential intracellular delivery of pDNA payloads, and block the CD44-angiogenic signaling for pursuit of inhibited tumorigenesis, leading to effective ablation of primary tumor and lung metastasis. Besides, the endocytosis procedures could be visible with probe-loaded, intracellular pH-sensitive nanocarriers. For example, the endocytic pH-sensitive nanoparticles has been reported, which could specifically probe early endosomes or late endosomes/lysosomes with different pH-sensitive groups 197, 198, and even probe early endosomes (pH 6.0) at single-organelle resolution 199. Moreover, the intracellular pH could trigger controlled drug release from nanocarriers 200-203. With one example, the cRGD-decorated polymeric micelles that self-assembled from epirubicin- conjugated block copolymers through hydrazide bonds, could specifically delivery and release epirubicin inside cancer cells for effective tumor ablation 204.

**Figure 9:**
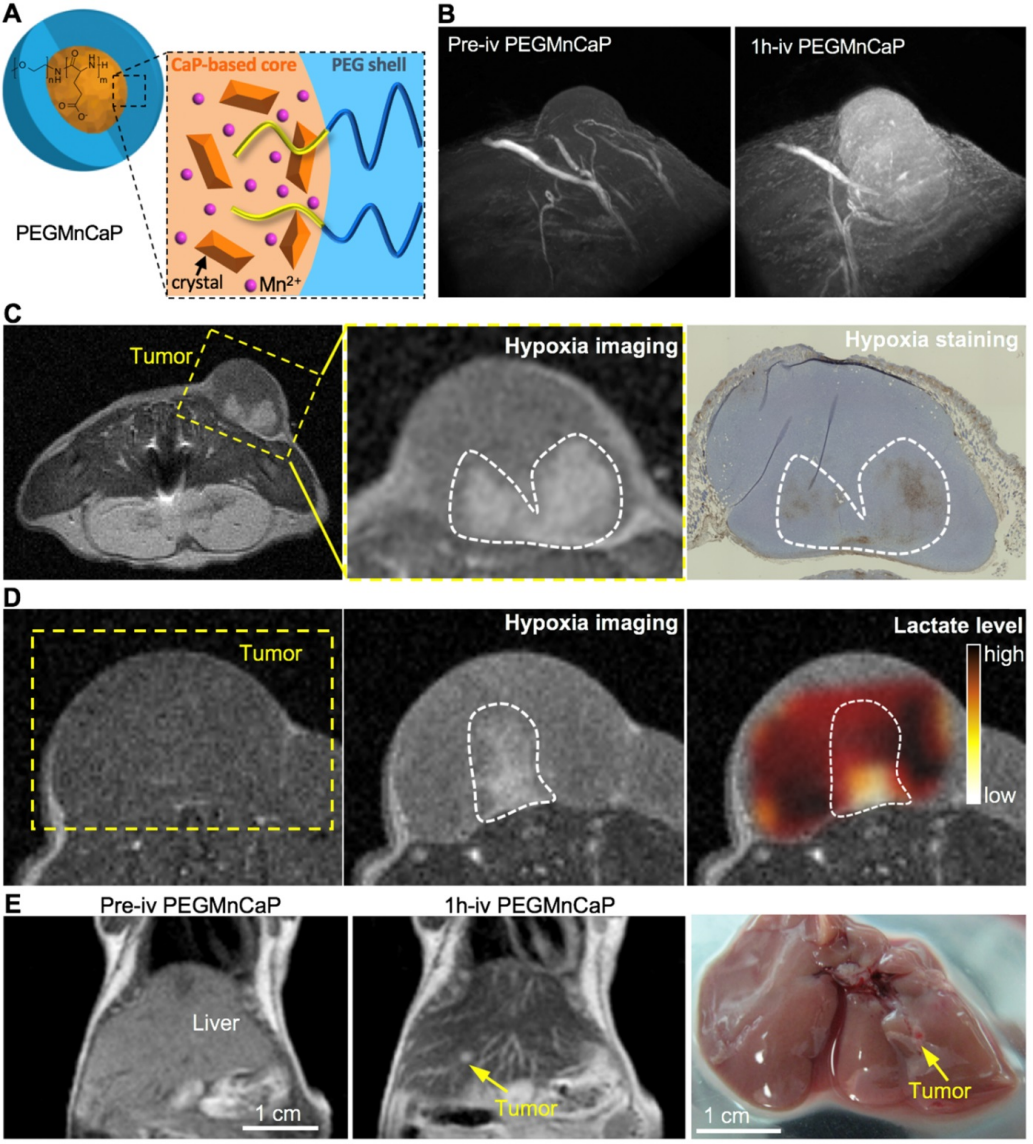
** The pH-responsive PEGMnCaP nanocarriers with contrast amplification ability have been developed for MR imaging of tumor malignancy. (A)** The composition and characterization of Mn^2+^-doped PEGMnCaP. **(B)** PEGMnCaP specifically enhanced the contrast in C26 tumors for three-dimensional (3D) MR imaging. **(C,D)** PEGMnCaP probed hypoxia in tumors as confirmed by immune-staining of hypoxia (C) and chemical shift imaging (CSI) of lactate (D). **(E)** PEGMnCaP for precisely MR imaging of 1-2 mm ultra-small metastasis in liver. Adapted with permission from ref. 205, copyright 2016 Springer Nature Limited.

**Figure 10:**
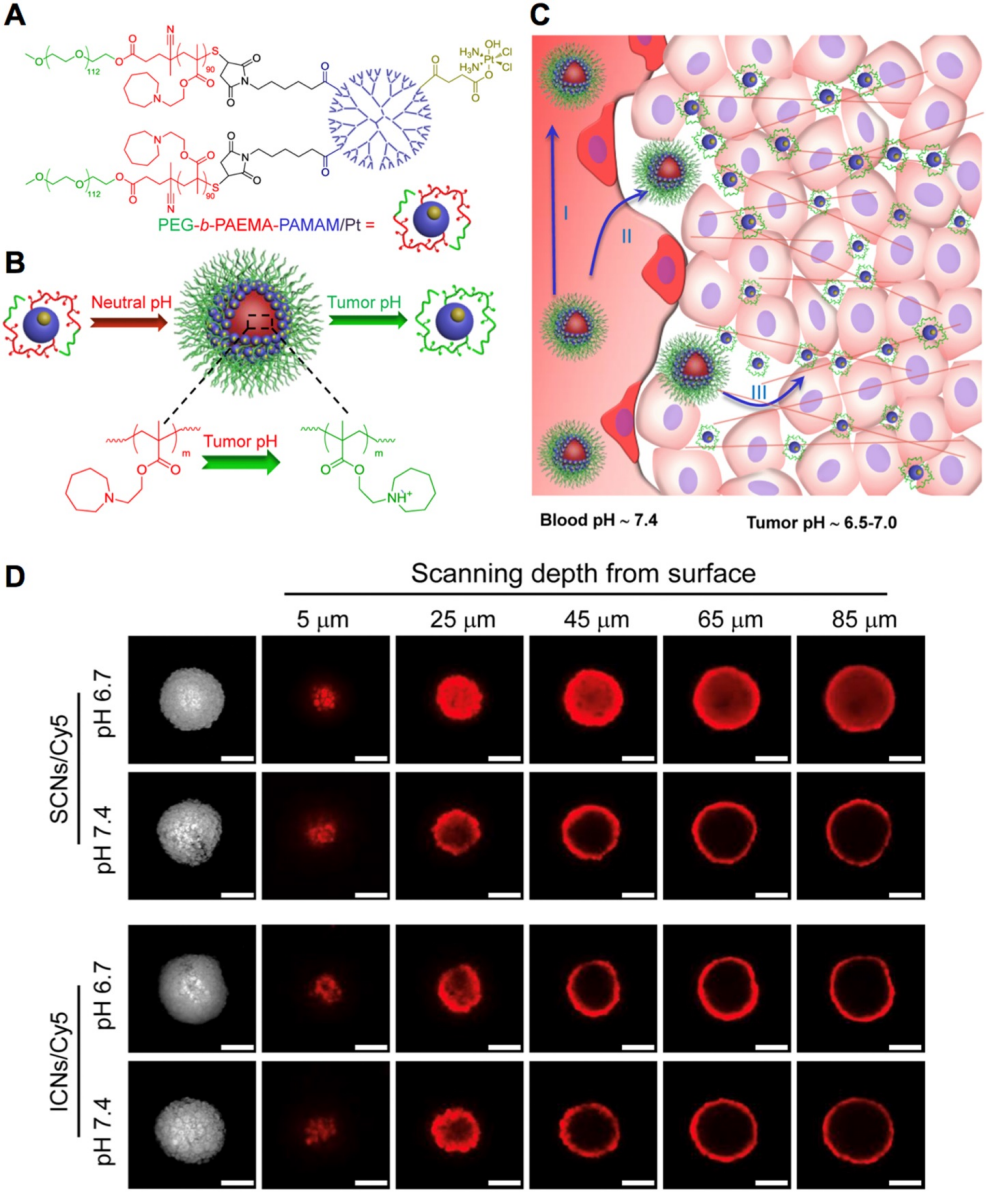
** The pH-responsive nanocarriers for tumor therapy. (A)** The structure of pH-sensitive polymer-drug conjugates. **(B)** Illustration of pH-dependent self-assembly and disassociation of PEG-*b*-PAEMA-PAMAM/Pt nanocarriers (SCNs/Pt) at different pH. **(C)** Illustration of pH-triggered disassociation of SCNs/Pt nanocarriers in tumors. **(D)** The penetration of SCNs/Pt nanocarriers in BxPC3 pancreatic cancer spheroids. Adapted with permission from ref. 211, copyright 2016 American Chemical Society.

Functional nanocarriers could also response to the low pH in tumor microenvironment for cancer- specific theranostics. Firstly, the pH-sensitive nanocarriers could incorporate different types of imaging probes for tumor-selective imaging and diagnosis. For instance, the pH-sensitive polymeric micelles incorporating fluorescence dye could specifically probe several types of solid tumors, due to the specific exposure of dyes in tumors, while the diagnostic selectivity could be promoted higher by installing targeting moieties (*i.e.*, cRGD) on the surface of micelles 20. The nanocarriers could further be utilized for fluorescence imaging-guided surgical resection of tumors 206. Considering the limited penetration of optical imaging, the pH-sensitive nanocarriers have been exploited for tumor imaging by MRI 207, 208. For instance, the Mn^2+^-doped, polymer hybrid CaP nanocarriers (PEGMnCaP) have been developed with intratumoral pH-triggered contrast amplification for MR imaging of tumor malignancy (Figure 9A), as the released Mn^2+^ could bind to surrounding proteins to boost much higher relativities. It could specifically and sensitively amplify the contrast in tumors for accurate two- and three-dimensional MR imaging (Figure 9B). The PEGMnCaP could also distinguish hypoxia in tumors with even higher contrast enhancement than the surrounding tumor regions, as more Mn^2+^ were released in hypoxic regions with lower pH, while the hypoxia imaging was confirmed by immunostaining of hypoxia (Figure 9C) and checking the lactate level in the detected hypoxia regions (Figure 9D). It further accurately probed ultra-small liver metastasis (Figure 9E), which was difficult to be detected by conventional CAs. The pH-triggered MR imaging of solid tumors could be further applied for imaging-guided tumor neutron capture therapy 165. For example, the pH-sensitive block copolymer hybrid CaP nanocarriers further demonstrated high performance in cancer theranostics by incorporating Gd-DTPA for tumor diagnosis and promoted gadolinium neutron capture therapy (GdNCT) 165, 208. Besides, the intratumoral pH could also trigger size switching for improved penetration of nanocarriers 186, 209, as comparable large size of nanocarriers benefits long circulation, while small size benefits intratumoral penetration 6, 210. For instance, the polymeric micelles have been self-assembled with platinum (Pt)-drug conjugated, pH-sensitive poly(ethylene glycol)-*b*-poly(2-azepane ethyl methacrylate)-modified polyamidoamine dendrimers (PEG-*b*-PAEMA-PAMAM/Pt) (Figure 10A). It could be disassociated into small size of polymer-drug conjugates by responding to tumor pH for deep penetration in tumors, exhibiting improved therapeutic efficacy (Figure 10B-D) 211. Moreover, nanocarriers could response to pH for surface charge conversion in tumor microenvironment 212, 213, as neutral or negative charged nanocarriers holds the “stealth effect” during long circulation, while positive charged nanocarriers are more likely to internalize with cancer cells. Regarding this point, the surface of polymeric micelles were designed to switch from neutral charge at blood pH 7.4 to cationic at tumorous pH 6.5, which could maintain their “stealth effect” during circulation and increase internalization with cancer cells for improved tumor accumulation 195. By tumor pH-triggered surface conversion, nanocarriers could also be applied for tumor-specific molecular imaging 214. In addition, by conjugating ligands (*e.g.*, biotin) to tumor pH-sensitive polymers, it was applied to hide the targeting ligands inside the PEG shell during circulation (*i.e.*, pH 7.4) and present ligands in tumor microenvironment (*i.e.*, pH <7.0) 215, to avoid unspecific internalization and uptake of ligands during circulation, as well as improve tumor active targeting efficacy 216. The ligand- installed, pH-sensitive nanocarriers were reported to target tumors and spontaneous metastasis with effectively suppressed tumor growth 202.

### Hypoxia-responsive nanocarriers

The poorly vascularization inside solid tumors is likely to form hypoxia (low oxygen level), which plays an important role in cancer progression, such as locoregional spread and distant metastasis 217. The promoted malignant phenotype by hypoxia has negative impact on prognosis and therapy and leads to resistance to standard therapy (*e.g.*, radiotherapy, chemotherapy). Therefore, several strategies have been utilized for treating hypoxic tumors, mainly including increasing the oxygen level and using hypoxia activatable prodrugs, *etc* 218. Until now, several types of nanocarriers have been engineered for drug delivery to hypoxic tumors (Table 5) 219, including liposomes 220, silica nanoparticles 221, upconversion nanoparticles (UCNPs), layer-by-layer nanoparticles 222, nanovesicles 128, polymeric micelles 223, polymersomes 224, albumin nanoparticles 225, cell membrane coated metal organic framework (MOF) 226, solid-state sensors 227, polymeric probes 228, and polymer hybrid CaP nanoparticles 205, *etc.* Meanwhile, different cargos could be loaded inside the hypoxia-activation nanocarriers, ranging from imaging agents (*e.g.*, contrast agents), prodrugs (*e.g.*, dihydrochloride (AQ4N)), anticancer drugs (*e.g.*, doxorubicin), siRNA and photosensitizers (*e.g.*, ICG), *etc.*, demonstrating high performance in hypoxic tumor imaging and effective therapy by overcoming drug resistance 229.

**Figure 11:**
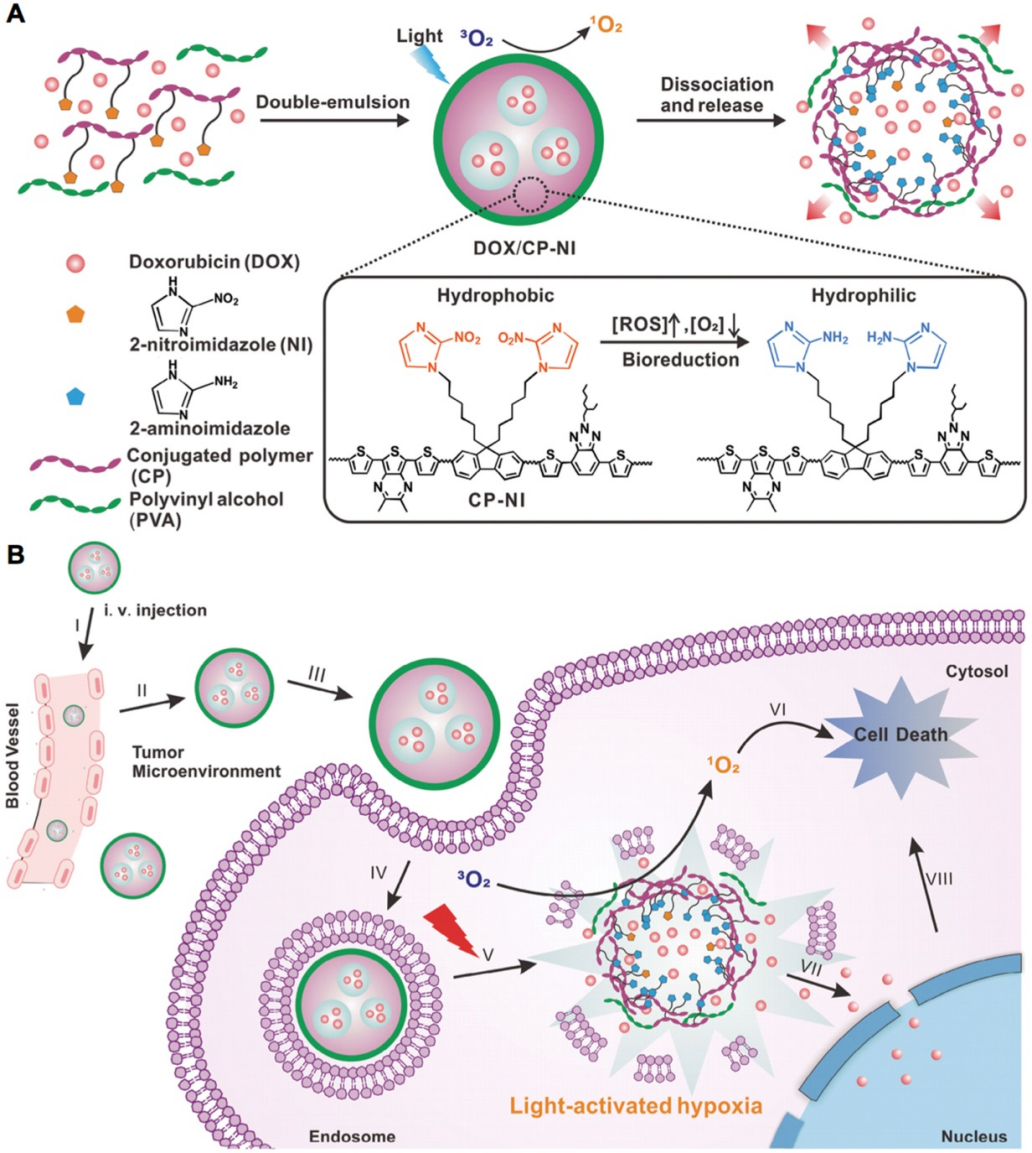
** Schematic illustration of light-activated hypoxia-responsive nanocarriers. (A)**Preparation of nanocarriers. **(B)**Nanocarriers generated ROS to induce local hypoxic environment, which triggered drug release to enhance the synergistic anticancer efficacy. Adapted with permission from ref. 233, copyright 2016 WILEY-VCH Verlag GmbH & Co. KGaA, Weinheim.

The tumor hypoxia could be targeted with hypoxia-responsive and some pH-sensitive nanocarriers, since hypoxic tumor regions are generally associated low pH due to the glycolysis of glucose and production of H^+^ and lactate 237. The major strategy is utilizing hypoxia-sensitive nanocarriers, which are generally constructed with hypoxia- sensitive materials or derivates, *e.g.*, 2-nitroimidazole 238-240, nitroimidazole 241-243, metronidazole 236, azobenzene 244-246, nitro-benzene derivatives 223 and iridium (III) complexes, *etc*. Hypoxia could trigger cargo release from the hypoxia-sensitive nanocarriers, *e.g.*, the incorporated antibody (*i.e.*, Cetuximab) could be released from the silica nanoparticles in hypoxic tumors due to the cleavage of the hypoxia-sensitive cross-linkers (*i.e.*, Azo monomer) 231. In another study, the nanocarriers were prepared with hypoxia-sensitive 2-nitroimidazole and light-sensitive conjugated polymers for generating ROS and local hypoxia after laser irradiation, to trigger doxorubicin release for enhanced synergistic anticancer efficacy (Figure 11) 233. The hypoxia- sensitive nanocarriers also facilitate molecular imaging of tumors and metastasis. For example, the nanoscale probes with oxygen level-sensitive iridium (III) complexes have demonstrated high potential for optical imaging of tumors and metastatic lesions 228, 247. Besides, some nanocarriers could delivery hypoxia-activatable prodrugs [*e.g.*, tirapazamine (TPZ) and banoxantrone (AQ4N), *etc*.] to hypoxic tumors for enhanced therapy, while some photosensitizers could be co-loaded to generate hypoxia by laser irradiation for prodrug activation. For instance, the ICG and TPZ-incorporated liposomes with iRGD as targeting moieties could target both normoxic and hypoxic cancer cells, while the irradiation of ICG by NIR laser could produce extra hypoxia activate TPZ for enhanced therapy 232. In another example, the vessel-disruptive agents (*i.e.* 5,6-dimethylxanthenone- 4-acetic acid) and TPZ incorporated, platelet membrane-coated nanoparticles could disrupt tumor blood vasculatures to promote drug accumulation for improved hypoxia-sensitive therapy 248. In addition, some pH-sensitive nanocarriers have also be applied for treating tumor hypoxia 249, *e.g.*, the pH-sensitive nanoparticles formed by layer-by-layer procedure could target hypoxic tumors for fluorescence imaging with the incorporated QDs 222. So far, the hypoxia- sensitive nanocarriers have exhibited much progress in drug delivery to hypoxic tumor for molecular imaging and improved therapy. However, some underlying problems would be addressed in future studies, such as modulating hypoxic tumor microenvironment, increasing drug penetration and oxygen level, and clinical translation of hypoxia-responsive nanocarriers.

### Redox-responsive nanocarriers

The redox-responsive nanocarriers have been widely applied for drug delivery due to the significantly different reduction potentials and capacities in tumors,* e.g.*, the glutathione (GSH) level inside cancer cells (2-10 mM) is remarkable higher than that in normal regions (2-10 μM). Until now, several redox-sensitive nanocarriers have been engineered (Table 6), including nanocapsules 250, mesoporous silica nanoparticles 251, polymer-drug conjugates 252, polymersomes 253, polymeric vesicles 254, polymeric micelles 255-257, nanogels 258, gold nanoparticles 259 and hybrid nanoparticles 260, *etc*. The disulfide bonds could be cleaved into sulfhydryl groups by GSH 261, while the diselenide bonds (Se-Se) are also sensitive to redox potential 262, but with lower bond energy than that of disulfide bonds 263. Moreover, the H_2_O_2_-responsive nanocarriers have also been developed for tumor therapy 264, 265, including for treating hypoxic tumors 266 and multidrug resistant tumors 267.

**Figure 12:**
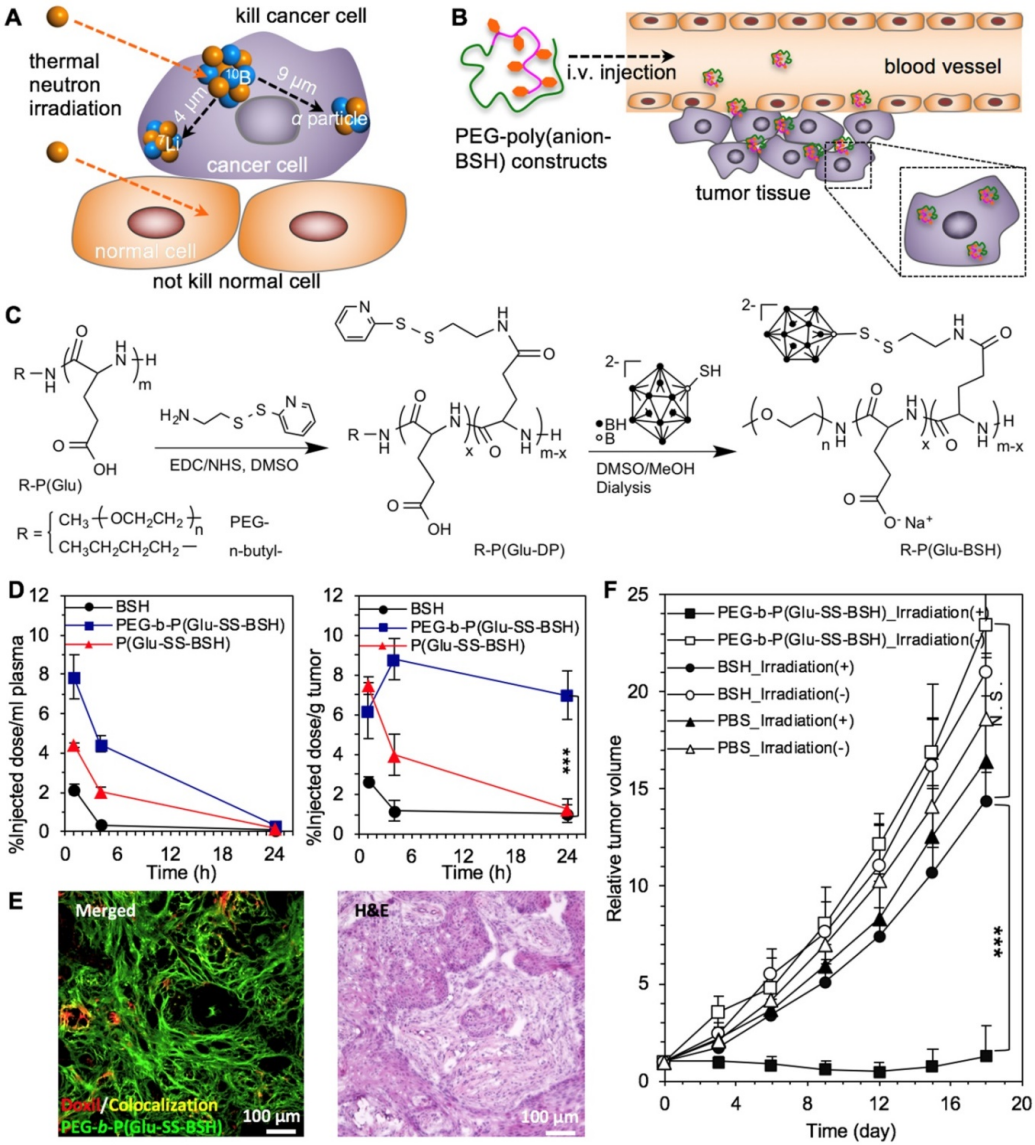
** The redox-responsive nanocarriers for drug delivery to tumors toward effective therapy. (A,B)** Illustration of boron neutron capture therapy (A) and nanocarriers for tumor BNCT (B). **(C)**The synthesis of redox-responsive polymeric nanocarriers. **(D)** Plasma clearance and tumor distribution of BSH and BSH-polymer conjugates. **(E)** The deep penetration of BSH-polymer conjugates in BxPC3 pancreatic tumors. **(F)** Boron neutron capture therapy of solid tumors with the polymer-boron cluster conjugates. Adapted with permission from ref. 252, copyright 2017 Elsevier B.V.

The redox-sensitive nanocarriers could trigger cargo release inside cancer cells 268, as some bioactive compounds were conjugated to nanomaterials through the disulfide bonds 252, 269 and the drug-loaded cavities in some nanocarriers (*e.g.*, mesoporous silica nanoparticles) were sealed by disulfide bonds 251. The redox-sensitive strategy could also be applied to detach the surface shell 270, and cross-link the core to increase the stability of nanocarriers 271, 272. In another strategy, the cationic vesicles were formed by chelating of Fe^3+^ with amphiphilic piliararene, exhibiting GSH-triggered release of incorporated doxorubicin and siRNA from the collapse vesicles, as a result of GSH-induced reduction of Fe^3+^ to Fe^2+^ inside cancer cells 254. Besides, the redox-responsive function could trigger the disassociation and degradation of nanocarriers inside cancer cells, as some nanocarriers were cross-linked by redox-sensitive bonds to increase the stability 271, 273. The disulfide bonds cross-linked polymer nanocapsules could be disassociated by responding to GSH and dithiothreitol (DTT) 250. Meanwhile, nanocarriers prepared by polymers with diselenide bonds (Se-Se) could also response to environmental redox-potential (*i.e.*, GSH, H_2_O_2_) for controlled disassociation of nanoparticles and release of cargos 263. Moreover, the redox-responsive nanocarriers facilitate intracellular delivery of bioactive compounds into cancer cells to overcome the cellular barriers, such as siRNA 254 and sodium borocaptate (BSH) 255, *etc.* For one example, the BSH-polymer conjugates have been engineered by conjugating with disulfide bonds for tumor boron neutron capture therapy (BNCT), because of the poor cellular uptake of clinically approved ^10^B-compounds (*e.g.*, BSH) and the limited effective distance almost within diameter of cancer cells (Figure 12A-C) 252. The BSH-polymer conjugates have significantly promoted the intracellular delivery of BSH, slightly extended the half-life in blood circulation and highly enhanced the tumor accumulation for deep penetration in tumor tissues and significant tumor therapy by BNCT (Figure 12D-F). Furthermore, the morphology of redox-sensitive nanocarriers may affect the intracellular delivery of cargos. Therefore, nanocarriers with different morphologies have been self-assembled with camptothecin and polymers through the disulfide bonds, including spheres, smooth disks, vesicles, and staggered lamellae 274, while the staggered lamellae ones demonstrated the most efficient cellular internalization than others. In addition, the redox-responsive nanocarriers demonstrated high potential for treating hypoxia tumors. For example, the Cy5.5-deoxybouvardin (RA-V) conjugates incorperated nanocarriers could target cancer cells by cRGD ligands, as well as release RA-V for intracellular fluorescence imaging and inducing apoptosis of cancer cells 266.

### Enzyme-responsive nanocarriers

Enzymes play an important role in biological reactions, while the unregulated expression of certain enzymes in neoplastic conditions could be triggers for enzyme-responsive drug delivery. Several enzyme- responsive nanocarriers have been engineered for achieving controlled release of cargos in tumors and cancer cells 280, 281, prodrug/ligands activation, as well as morphology change, mainly including mesoporous silica nanoparticles 282, 283, dendrimers 284, magnetic nanoparticles 285, 286, polymeric micelles 287 and liposomes 288, 289 *etc.* As shown in Table 7, nanocarriers could response to several upregulated enzymes in tumor microenvironment and cancer cells 290, which are mainly including oxidoreductases (*e.g.*, peroxidases) 291, transferases (*e.g*., creatine kinase) 289, and hydrolases, such as matrix metalloproteinases (MMPs) 292-294, human recombinant caspase 3 295, proteinase K 60, 296, intestinal protease 286, cathepsin B 297 and trypsin 298, 299 *etc*.

The enzyme-sensitive nanocarriers could be utilized in the following aspects: (1) Activating prodrugs, probes and ligands by cutting the enzyme-sensitive bonds between the bioactive compounds and protective groups; (2) Degradation or disassociation of nanocarriers through enzyme- triggered cleavage of polymer backbones, charge conversion of nanomaterials and disassembly of nanoparticles; (3) Direct cleaving the conjugation between nanocarriers and drugs; (4) Enzyme- triggered physical disruption of nanocarriers; (5) Enzyme-triggered controlled release of cargos. For achieving enzyme-sensitive function, several factors should be considered for rational design nanocarriers: (1) The recognition and accessibility of enzymes to the sensitive groups/substrates in nanocarriers; (2) The threshold of the substrates that responding to enzymes, which should ensure the enzyme-triggered reaction; (3) the influence of physiological conditions and the physicochemical properties to the enzyme- sensitivity.

**Figure 13:**
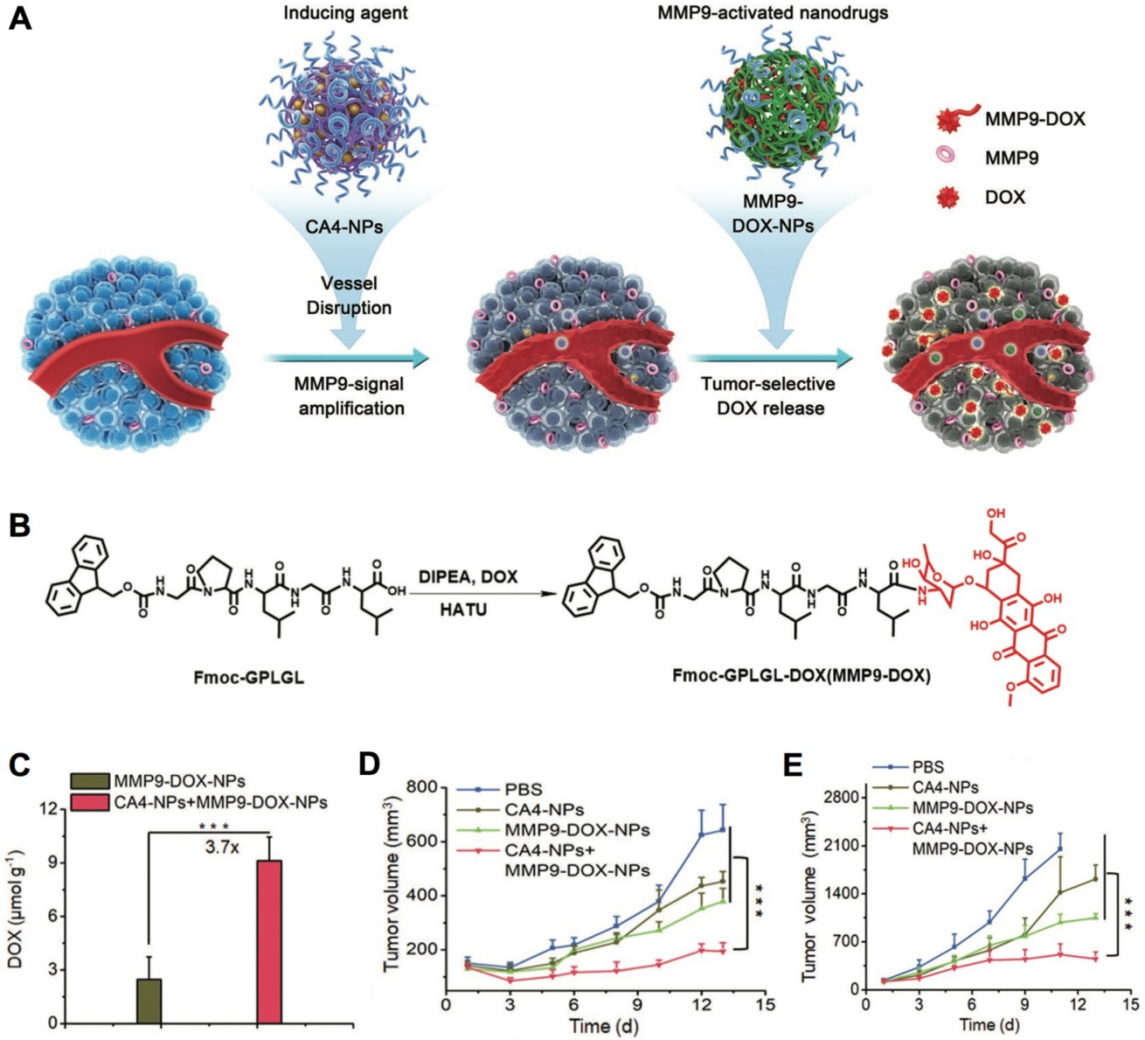
** Enzyme-responsive nanocarriers for cancer therapy. (A)** Schematic illustration of nanocarriers incorporating combretastatin A4 nanodrug (CA4) plus MMP9-activatable doxorubicin prodrug for tumor therapy. **(B)** The chemical structure of MM9-activatable MMP9-activated doxorubicin prodrug. **(C)** The distribution of doxorubicin in tumors. **(D,E)** Tumor inhibition rate in 4T1 (D) and C26 (E) tumor models. Adapted with permission from ref. 300, copyright 2019 John Wiley & Sons, Inc.

The specific enzyme-triggered cargo release allows drug delivery to tumors and avoids cargo exposure during circulation, which could maintain the activity of bioactive compounds, while avoid causing sides effects to normal organs/tissues. For enzyme-triggered drug release, the cathepsin could cleave the hydrolyze peptide bonds in gemcitabine- conjugated dendrimer nanocarriers inside lysosomes to liberate gemcitabine and cationic dendrimers, leading to lysosome escape and intracellular gemcitabine delivery 284. In another study, the hyaluronic acid coated and prodrug-loaded nanoparticles could specifically release paclitaxel inside cancer cells by affecting the hydrolyze peptide bonds with human recombinant caspase 3 295. Besides, the prodrugs/ probes could be activated by enzymes in tumors, as the prodrug strategy is generally applied to protect the activity of drugs, probes and ligands during circulation to increase the diagnostic or therapeutic specificity 301. In one example, the protease- activatable nanoprobes have been developed by combining fluorescent dye and Fe_3_O_4_ nanocrystals through MMP-9 302, which could turn “ON” the fluorescence for tumor imaging when the peptide substrates linkers were cleaved by protease. In another case, the MMP9-activatable doxorubicin prodrug-loaded nanocarriers were developed (Figure 13A,B) 300, to combine with combretastatin A4 (CA4)-loaded nanocarriers for cancer synergistic treatment. The CA4-loaded nanocarriers could disrupt tumor blood vasculature and selectively enhance MMP9 expression in tumors to promote the accumulation of doxorubicin (Figure 13C), leading to effective treatment of 4T1 and C26 tumors (Figure 13D,E). Moreover, the enzyme-responsive nanocarriers could be applied for tumor specific imaging, e.g., the MMP-responsive iron oxide nanoparticles have specifically enhanced the *T*_2_-weighted contrast in tumors for diagnosis by MRI 285. Furthermore, the enzyme could uncap the surface shell (*e.g.*, peptides) of nanocarriers to improve their accumulation in tumors. For example, the nanocarriers self- assembled by paclitaxel- conjugated block copolymers and enzyme-recognition peptide shell, could change the morphology due to the cleavage of peptide shell by MMP, leading to high accumulation of the polymer-drug conjugates in tumors 292. In addition, the enzyme-responsive function could be applied for disassociation of nanocarriers. The azobenzene-linked amphiphilic diblock copolymers have been applied to form polymeric micelles, and micellar architecture could be disrupted by responding to azoreductase and nicotinamide adenine dinucleotide phosphate (NADPH) 303. It demonstrated high potential in the arena of colon-specific drug delivery, as azoreductase is existed in human intestine. The enzyme-triggered degradation of nanocarriers into small size structures would improve the penetration of drug delivery systems throughout the tumor's interstitial spaces. For instance, the 100 nm nanoparticles could be reduced to 10 nm by responding to proteases (*i.e.*, MMP-2) in tumor microenvironment, which effectively enhanced the diffusion of drugs into the tumor's dense collagen matrix, while maintained long circulation for achieving EPR effect 304. Overall, the enzyme- sensitive nanocarriers have demonstrated high potential in tumor diagnosis 285, 286, as well as treating primary and metastatic tumors 293, 294, 305.

## Multimodal-responsive nanocarriers

**Figure 14:**
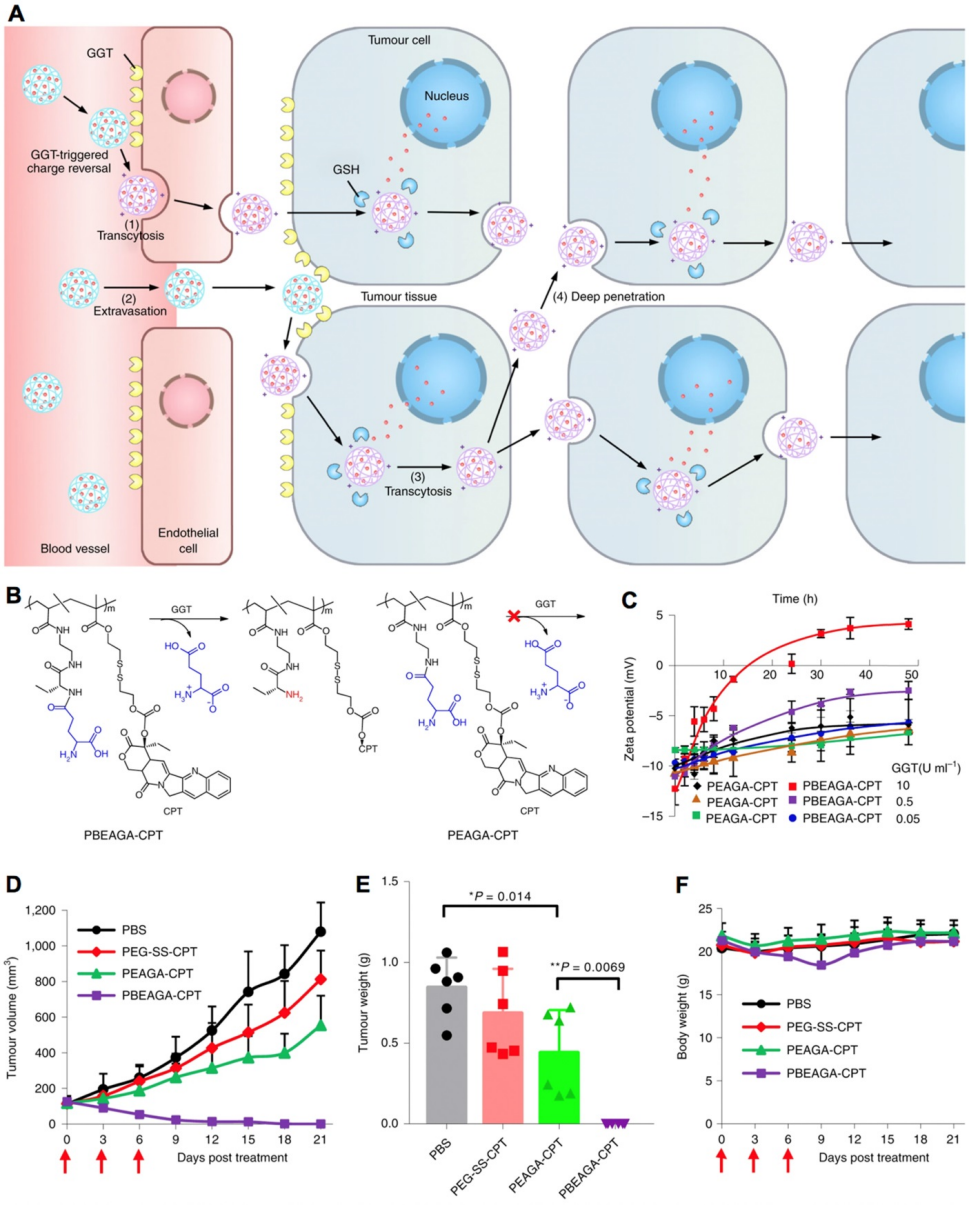
** Multimodal-responsive polymer-drug conjugated nanocarriers. (A)** Illustration of the cationization-initiated transcytosis-mediated tumour penetration for transendothelial and transcellular transport of nanocarriers. **(B)** The structures of GGT-responsive cationizing PBEAGA-CPT conjugates and the non-GGT-responsive PEAGA-CPT conjugates.** (C)** The zeta potentials of the nanocarriers. **(D-F)** Antitumor efficacy of polymer-drug conjugated nanocarriers against subcutaneous HepG2 tumors, where the tumor growth rate (D), tumor weight (E) and bodyweight (F) were measured. Adapted with permission from ref. 322, copyright 2016 Springer Nature Limited.

In addition, nanocarriers have also been engineered with multiple stimuli-responsive functions, facilitating multistage drug delivery, as well as achieving higher specificity and efficacy. For example, nanocarriers responding to both intracellular pH and GSH have been developed for promoted intracellular drug delivery 314. In another study, the developed platinum drug delivery nanocarriers could response to intracellular GSH for disassociation, and response to intracellular low pH for controlled drug release 277. Indeed, the multiple stimuli-responsive nanocarriers hold high potential in achieving long circulation, high tumor accumulation, deep penetration in tumor tissues, internalization with cancer cells and endosome escape, *etc*. Thus, several multiple stimuli-responsive nanocarriers have been engineered for delivery cargos to tumors 315-321. In one example, the multiple stimuli- responsive nanocarriers could be discharged into small nanoparticles by responding to the low pH in tumor microenvironment, and then the platinum prodrugs in the small nanoparticles were activated by GSH for promoted penetrating and treating the poorly permeable pancreatic tumors 209. In another example, the nanocarriers made by γ-glutamyl-based polymer-drug conjugates (PBEAGA-CPT) conjugates could response to both γ-glutamyl transpeptidase (GGT) and GSH have been developed 322, which could convert to be positive charged nanomaterials by responding to GGT for internalization with cancer cells and by responding to GSH inside cancer cells to release CPT (Figure 14A-C). The multimodal responsive polymer-drug conjugated nanocarriers have demonstrated high efficacy in transcytosis, extravasation, internalization with cancer cells and deep tumor penetration, leading to effective supression of subcutaneous HepG2 tumors (Figure 14D-F). In general, it is sophisticate for developing multiple stimuli-responsive nanocarriers, and also difficult to maintain the multiple functions in biological systems. Thus, nanocarriers with single or dual stimuli- responsive functions have been more focused 49, 323. For instance, the polyphosphazene nanocarriers with pH- and redox-sensitivities have been engineered for tumor multimodal imaging- guided chemo-photodynamic therapy 324-326. Here nanocarriers for multiple stimuli-triggered drug delivery were briefly introduced, as each stimuli- responsive function has already been discussed above.

## Clinical translation of the stimuli-responsive nanocarriers

The advances in stimuli-responsive nanocarriers have led to clinical translation of several formulations. As shown in Table 8, there are six nanocarriers responding to magnetic, temperature, pH and secretory phospholipase A2 (sPLA2), are under clinical translation. Two magnetic-sensitive iron- based nanocarriers, iron oxide magnetite, and doxorubicin-loaded iron and carbon (MTC-DOX), are under clinical trial for treating cancers. The iron oxide magnetite was conducted Phase I clinical trial to evaluate safety, retention and distribution after injection, which final score is for treating prostate cancer in men by thermal ablation. Three clinical trials have been applied for MTC-DOX, including Phase II and III studying the safety, tolerance and efficacy (survival time) on treating unresectable hepatocellular carcinoma (NCT00034333); Phase I and II evaluation of prohibiting hepatocellular carcinoma progression after injection with external magnet (NCT00054951); and Phase I and II studying on liver metastasis (NCT00041808). Besides, the thermal-sensitive doxorubicin- incorporated liposomes (ThermoDox) have been applied for the following three clinical studies: Phase I and II studying the maximum tolerated dose, safety, pharmacokinetics and hyperthermia effects in patients with recurrent regional breast cancer (NCT00826085); Phase I investigation of doxorubicin release from liposome by focused ultrasound in liver tumors (NCT02181075); and MRI and high intensity focused ultrasound (HIFU) combined study to determine doxorubicin release in pediatric refractory solid tumor (NCT02536183). The clinical trial of ThermoDox has also be designed to evaluate the safety and efficacy by combining with HIFU on several tumors (Phase II, NCT01640847), *e.g.*, painful bone metastases, breast carcinoma, non-small cell lung cancer, small cell lung cancer and adenocarcinoma; as well as study the efficacy on treating hepatocellular carcinoma combined with standardized radiofrequency ablation (Phase III, NCT02112656). Moreover, the pH-responsive, epirubicin-loaded polymeric micelles (NC6300) have entered Phase I and II study (NCT03168061) for evaluating the dose, activity and tolerability in patients with soft tissue sarcoma. In previous preclinical clinical study, NC6300 could reduce the cardiotoxicity of epirubicin by conjugating to polymers through pH-sensitive bonds (*i.e.*, hydrazone) 327, and exhibited better therapeutic effect (10 mg/kg based on epirubicin) on treating hepatocellular carcinoma 328. The preclinical evaluation has provided positive evidences for further clinical evaluation. In addition, the secretory phospholipase A2 (sPLA2)-sensitive, cisplatin- incorporated liposomes (LiPlaCis) have entered Phase I and II to study the safety, tolerability and sensitivity on patients with advanced breast cancer and metastatic breast cancer (NCT01861496). Although with progress, the clinical translation of stimuli- responsive nanocarriers still encountered several barriers: (1) the differences between animal tumor models and tumors in patients, as tumors in patients are more heterogeneity and complicated; (2) the toxicity, biosafety and biodegradability of nanocarriers should be addressed; (3) the stable stimuli-responsive function *in vivo*; (4) the tumor accumulation and therapeutic efficacy of stimuli- sensitive nanocarriers should be proved in clinical trial; (5) the factors that influence the stimuli- responsive properties *in vivo* should be clarified; (6) the right dose and administration way should be studied, *e.g.*, intravenous injection (*i.v.*), intraperitoneal injection (*i.p.*). Therefore, future work would focus on clinical translation of the stimuli-sensitive nanocarriers, and optimizing the formulations from lessons of clinical trial.

## Conclusion

The nanocarriers bring novel strategy for delivery bioactive compounds to tumors. The stimuli-sensitive nanocarriers provide high specificity and multiple functions in drug delivery, including controlled release, alerted tumor accumulation, switch “ON-OFF” activities, as well as promoted diagnostic and therapeutic accuracy and efficacy. Besides, the rational design of stimuli-nanocarriers has considered their biological manners in tumor microenvironment and cancer cells to maximize the efficacy and minimizing the adverse effects to normal organs and tissues. Until now, numerous external and internal stimuli-sensitive nanocarriers have been developed, exhibiting better outcomes than the conventional formulations. The stimuli-responsive systems could be widely applied for diagnosis, probing, sensing and therapy tumors and other diseases, such as cardiovascular diseases, *etc*. Moreover, maintaining the stimuli-sensitivity in large scale produced nanocarriers would be potential challenge. Furthermore, although with extensive studies on stimuli-sensitive nanocarriers, only a few formulations have entered clinical translation, which requires future extensive works on clinical translation. In addition, considering the heterogeneity of tumors, the molecular imaging would be applied for screening the stimuli-responsive nanocarriers in tumors and patients, to predict and study the sensitivity and responses 329. Meanwhile, the stimuli-responsive nanocarriers may also be combined with antibodies for tumor immunotherapy 330, 331. Overall, the development of nanocarriers responding to external and internal stimuli in diseased regions would promote the advent of “magic bullets” for tumor precision diagnosis and therapy in future.

## Figures and Tables

**Table 1:** Representative ultrasound-responsive nanocarriers

Nanocarriers	Ultrasound-sensitive strategy/materials	Cargos	Applications	Ref.
Converting microbubbles	Converting porphyrin microbubbles to nanoparticles by ultrasound	Porphyrin and perfluorocarbon gas	Ultrasound imaging	33
CaCO_3_ nanoparticles	The CaCO_3_ could generate CO_2_ in the acidic tumor microenvironment	Doxorubicin	Tumor ultrasound imaging, drug release and tumor therapy	39
Nanobubbles	CO_2_ gas-generating polymeric nanoparticles	-	Ultrasound Imaging	34
Liposome	Perfluorocarbon for ultrasound-sensitive	Doxorubicin, gold nanospheres	Cancer imaging, photothermal-chemotherapy	60
Liposome	Containing NH_4_HCO_3_ to generate gas in tumors	Docetaxel and NH_4_HCO_3_	Dual ligand targeted triplex therapy, and ultrasound imaging	61
Nanorattles	Perfluoropentane for ultrasound-sensitive	Perfluoropentane	Ultrasound and photoacoustic imaging, photothermal therapy	48
Nanodroplets	Perfluorocarbon	ZnF_16_Pc, IR dye, perfluorocarbon	Tumor multimodal imaging and therapy	62
Gas vesicles	Genetically encoded gas nanostructures from microorganisms	Gas	Ultrasound and multimodal imaging, molecular biosensors	44

**Table 2:** Representative thermal-responsive nanocarriers

Nanocarriers	Thermal-sensitive strategy/materials	Cargos	Applications	Ref.
Liposomes	The incorporated NH_4_HCO_3_ could response to local hyperemia for drug release	Doxorubicin, NH_4_HCO_3_	Temperature-controlled drug release	64
Nanoscale vesicles	The temperature-sensitive leucine zipper peptide in the wall of vesicles could open pores for cargo release	Doxorubicin	Temperature-triggered drug release	87
Micelles	PMEEECL-*b*-POCTCL diblock copolymer displays phase transition at temperature above its LCST for cargo release	Nile Red, doxorubicin	Thermal-triggered drug release, efficient drug delivery to cancer cells	67
Nanogels	PNIPAM grafted chitosan nanogels response to temperature for drug release	Curcumin	Temperature-triggered drug release, intracellular drug delivery	73
siRNAsome	With siRNA-SS-PNIPAM to form vesicles responding to temperature higher than LCST	Doxorubicin, siRNA	Against multi-drug resistant cancer cells	78
Polymersomes	Thermal-sensitive PNIPAM gel in side pH-sensitive polymersomes	Doxorubicin	Dual-thermal, pH-responsive drug release, tumor therapy	88
Complexes	PEI-g-PMEOMA-*b*-PHEMA) polymers for temperature sensitive gene delivery	pDNA	Gene therapy of tumors	84
Nanocapsules	Forming Pluronic/PEI with high temperature to load siRNA, which could be released inside cancer cells with cold shock	siRNA	Enhanced intracellular siRNA delivery to HeLa cancer cells	72

**Table 3:** Representative magnetic-responsive nanocarriers

Nanocarriers	Magnetic-responsive strategy/materials	Cargos	Applications	Ref.
Multifunctional magnetic nanocarriers	Magnetic field guided tumor targeting of SPIOs-loaded nanocarriers	SPIOs, doxorubicin	Tumor-targeted therapy	95
Albumin nanocapsules	Magnetic guided tumor targeting	Fe_3_O_4_, hydrophilic drugs	Targeting cervical cancer cells	94
Magnetic nanoparticles	Nanoparticles response to the alternating magnetic field for geldanamycin release and effective apoptotic hyperemia to kill cancer cells	Geldanamycin, amine-functionalized Zn_0.4_Fe_2.6_O_4_	Nanoparticle-mediated resistance-free apoptotic hyperthermia for kill cancer cells	89
Mesoporous iron oxide nanoparticles	Burst gas generation and on-demand drug release upon high-frequency magnetic field exposure	Iron oxide nanoparticles, paclitaxel, perfluorohexane	Tumor active targeted thermos-chemo-therapy	107
Polymeric micelles	Generate magnetic hyperthermia and controlled drug release	La_0.7_Sr_0.3_MnO_3_, doxorubicin	Effective breast cancer theranostics	93
Multifunctional hybrid nanoparticle	Produce localized heat under an alternating magnetic field, which triggers the release of the loaded drug	Fe_3_O_4_, Au, carbon dots, doxorubicin	Photothermal therapy of melanoma tumor	115
Liposomes	Induce local hyperthermia by response to alternating magnetic field	Magnetic nanoparticles, rhodamine, photosensitizer	Ultimate hyperthermia and photodynamic therapy combined tumor ablation	91
Nanoparticles	Generate heat in response to an alternating current magnetic field	Fe_3_O_4_ nanoparticles, doxorubicin	Tumor active targeted therapy by magnetic hyperthermia and chemotherapy	116
Magnetic nanogels	Magnetic hyperthermia	Iron oxide nanoparticles, doxorubicin	Prostate cancer therapy by hyperthermia and chemotherapy	97
Porous magnetic microspheres	Produce thermal energy and trigger the vaporization of liquid perfluorohexane	Iron oxide nanoparticles, perfluorohexane	Tumor treatment by activating droplets vaporization	103
Magnetic nanoparticles	Localized hyperthermia kills tumor cell preferentially	Iron oxide nanoparticles	Treating primary and metastatic lung malignancies	109

**Table 4:** Representative light-responsive nanocarriers

Nanocarriers	Light-responsive mechanism/materials	Cargos	Applications	Ref.
Polyion complex vesicles (PICsomes)	Light-triggered release of photosensitizer, photochemical internalization	Al(III) phthalocyanine chloride disulfonic acid (AlPcS2a)	PDT of tumors, photoinduced cytoplasmic delivery of drugs	118
Three-layered polyplex micelles	Dendrimeric photosensitizer for light-responsive endo-/lysosomal escape	pDNA, photosensitizer	Light-induced systemic gene transfer for tumor therapy	119
Micelles	Using NIR light excitation of UCNPs to trigger dissociation of micelles	NaYF_4_:TmYb UCNPs	NIR light-triggered cargo release	123
Nanoparticles	Spiropyran for UV-Vis light responsive	Rhodamine B, coumarin 6, calcein, Cy5, paclitaxel, docetaxel, doxorubicin	Light-triggered drug delivery and tissue penetration	121
Nanoparticles	Photosensitizer Ce6 for light- triggered size reducing, and generation of O_2_^1^ (ROS)	Camptothecin, Ce6	Enhanced tumor penetration for combined therapy	159
Liposome	Porphyrin for light-responsive phototherapy	Doxorubicin, porphyrin	Chemotherapy and phototherapy of tumors	129
Lanthanide-doped UCNPs	Dithienylethene photo-responsive molecules	Er^3+^*/*Yb^3+^ and Tm^3+^/Yb^3+^ doped NaYF4 UCNPs	NIR light remote-control to drive the reversible photo-switching reactions	125, 126
Cell membrane-based nanocarriers	Indocyanine green (ICG) for photothermal therapy	Doxorubicin, ICG	NIR-triggered drug release and tumor active targeted photothermal and chemotherapy	160
Vesicle	The structure change of azobenzene makes disassociation with β-CD	β-CD, azobenzene	Mimic for cell aggregation	152
Nanogel	Graphene for light-triggered photothermal effects	Doxorubicin, graphene	Theranostics of lung cancer	131
Nanorods	Gold nanorods for thermal sensitivity	DNA, doxorubicin	Treatment of multidrug resistant cancer cells	134
Carbon nanotubes	Photothermal effects of carbon nanotubes	Doxorubicin	Photothermal and chemotherapy of tumor	138
2D transitional metal nanomaterials	Photothermal effects of MoS_2_	Doxorubicin	Photothermal and chemotherapy of tumor	139

**Table 5:** Representative hypoxia-responsive nanocarriers

Nanocarriers	Magnetic-responsive strategy/materials	Cargos	Applications	Ref.
Liposomes	The prodrug of banoxantrone dihydrochloride (AQ4N) could be activated in hypoxic environment caused by PDT	Ce6, AQ4N	Cancer therapy	230
Silica nanoquencher	Azo monomer; cell-penetrating poly(disulfide)s (CPD) coated silica nanoquencher (BS-*q*NP) (CPD-protein@BS-*q*NP)	Antibody (Cetuximab), fluorescent dye	Hypoxia-triggered protein release and fluorescence imaging	231
Upconversion nanoparticles (UCNPs)	Oxygen indicator [Ru(dpp)_3_]^2+^Cl_2_ for hypoxia detection as UCNPs provided the excitation light of [Ru(dpp)_3_]^2+^Cl_2_ by upconversion process at 980 nm	[Ru(dpp)_3_]^2+^Cl_2_, UCNPs	Imaging hypoxic regions or oxygen changes in cells and zebrafish	229
Nanoparticles	The photosensitizer of ICG-mediated PTT induced hypoxia, which then activated the prodrug of TPZ	TPZ, ICG	Tumor therapy by PDT and chemotherapy	232
Nanoparticles	The shift from hydrophobic to hydrophilic of 2-nitroimidazole that grafted to polymers in light-activated hypoxia	Doxorubicin, light-sensitive polymer	Hypoxia-triggered drug release, tumor	233
Nanoparticles	PEG-azo(azobenzene)-PEI-DOPE block copolymer	siRNA	siRNA delivery and tumor RNAi	234, 235
Nanoparticles	Layer-by-layer nanoparticles with a pH-sensitive layer for targeting of tumor hypoxia	Sulfonated polystyrene beads or carboxylated quantum dots	Systemic tumor targeting	222
Cancer cell membrane coated MOFs	The porphyrinic MOFs could generate toxic ROS for PDT and cause hypoxic regions for activating TPZ	Porphyrinic metal organic framework, TPZ	Tumor targeted PDT and chemotherapy	226
Nanovesicles	The light irradiation of Ce6 induced hypoxia for oxidation bioreduction of 2-nitroimidazole in polymers and activation of TPZ	Ce6, TPZ	Tumor fluorescence imaging and therapy	128
Polymeric micelles	The metronidazole (MN) grafted in polymers could change hydrophobicity in hypoxic conditions for drug release	Doxorubicin	Tumor chemotherapy and radiotherapy	236
Polymersomes	The PLA (polylactic acid)-azobenzene-PEG is sensitive to hypoxia	Gemcitabine, hypoxia- sensitive dye “Image-iT”	Tumor imaging and drug delivery	224
Albumin nanoparticles	With hypoxia-sensitive azobenzene linker to covalently bridge photosensitizer Ce6-conjugated HSA and oxaliplatin prodrug-conjugated HSA	Oxaliplatin prodrug, Ce6	Tumor chemotherapy and photodynamic therapy	225
Mesoporous silica nanoparticles	The Ce6-dopped mesoporous silica nanoparticles were decorated with PEG and glycol chitosan by hypoxia-sensitive azobenzene linker	Oligonucleotide (CpG), Ce6	Cancer immunotherapy	221
Solid-state sensors	Iodide-substituted difluoroboron dibenzoylmethane-poly(lactic acid) (BF_2_dbm(I)PLA) solid-state sensor material	BF_2_dbm(I)PLA	Tumor hypoxia optical imaging	227
Polymeric probes	Poly(N-vinylpyrrolidone)-conjugated iridium-(III) complex (Ir-PVP) and poly(ε-caprolactone)-*b*-poly(N- vinylpyrrolidone) (PCL-PVP) nanoparticles	Iridium (III) complex	Optical imaging of tumor and metastasis	228
Polymer hybrid CaP nanoparticles	Tumor pH-triggered release of Mn^2+^ from CaP to boost higher contrast enhancement in hypoxic tumor regions	Mn^2+^	MR imaging of solid tumors, hypoxia and metastasis	205

**Table 6:** Redox-responsive nanocarriers for cancer theranostics

Nanocarriers	Redox-responsive mechanism/materials	Cargos	Applications	Ref.
Nanocapsules	Disulfide bonds response to DTT) and GSH	Carboxyfluorescein	Redox-potential triggered drug release inside cancer cells	250
Mesoporous silica nanoparticles	Disulfide bonds	Fluorescence dye	Cell-specific targeting and redox-sensitive drug release	251
Mesoporous silica nanoparticles	Disulfide bonds	Doxorubicin	Controlled drug release and tumor active targeted therapy	275
Polymer-drug conjugates	Disulfide bonds	^10^B-based sodium borocaptate	Efficient tumor targeted therapy, deep penetration, GSH-triggered drug release	252
Polymeric vesicles	Oxidation of the central-block sulphide moieties to sulphoxides and ultimately sulphones by H_2_O_2_	-	The first example of use oxidative conversions to destabilize nanocarriers	276
Polymersomes	Disulfide bonds in poly (trimethylene carbonate-co-dithiolane trimethylene carbonate)	Doxorubicin	Lung cancer chemotherapy	253
Micelles	Disulfide bonds	Camptothecin	GSH-triggered drug release inside cancer cells for effective tumor therapy	124
Micelles	Se-Se bonds	Rhodamine B	GSH-triggered cargo release	263
Micelles	Disulfide bonds	siRNA	Cross-linked micelles with improved stability for siRNA delivery	271
Dendritic nanoparticles	Disulfide bonds	Cisplatin, fluorescence dye	Tumor theranostics	277
Cationic vesicles	Reduction of Fe^3+^ to Fe^2+^ by GSH	Anticancer drugs and siRNA	Redox‐responsive nanocarriers for drug/siRNA co‐delivery	254
Nanogels	Disulfide bonds	Camptothecin	Tumor therapy	258
Nanoparticles	Diselenide bonds	Paclitaxel	GSH-triggered drug release and tumor active targeted therapy	278
Nanoparticles	Catalase-response to H_2_O_2_	Catalase, photosensitizer of methylene blue	Light-triggered, H_2_O_2_-responsive release of cargos for treating hypoxic cancer cells	267
Polyphosphazene nanoparticles	Cross-linking by disulfide bonds	Doxorubicin	Redox-responsive chemotherapy and photothermal therapy	279

**Table 7:** Enzyme-responsive nanocarriers for cancer theranostics

	Bond type	Enzyme	Reaction	Occurrence	Materials	Cargo	Ref.
**Hydrolases**	Peptide bonds	α- Chymotrypsin	Hydrolyze peptide amide bonds	Pancreas	Hollow mesoporous silica/poly(L-lysine) particles	Fluorescein and cytosine-phosphodiester-guanine oligodeoxynucleotide (CpG ODN)	283
		Human recombinant caspase 3	Hydrolyze peptide bonds only after an aspartic acid residue	Cytoplasm	Hyaluronic acid coating caspase 3 loaded pure drug nanoparticles	Paclitaxel	295
		Cathepsin	Hydrolyze glycyl phenylalanyl leucyl glycine tetra-peptide	Lysosome	PEGylated lysine peptide dendrimer-gemcitabine conjugate	Gemcitabine	284
			Hydrolyze tetrapeptide glycyl phenylalanyl leucyl glycine tetra-peptide	Lysosome	Amphiphilic biodegradable triblock N-(2-hydroxypropyl methyl) acrylamide copolymer-gadolinium- paclitaxel-Cyanine5.5 conjugates	Paclitaxel	297
		Elastase	Hydrolyze peptide amide bonds of elastin	Tumor	PEGylated pDNA-nanoparticles	Nucleic acid	306
		MMPs	Hydrolyze peptide amide bonds of extracellular matrix proteins	Participate in tissue remodeling and metastasis	Low molecular weight protamine and conjugated it to PEG-PCL nanoparticles	Paclitaxel	307
			Hydrolyze peptide amide bonds of extracellular matrix proteins	Participate in tissue remodeling and metastasis	MSNs-Peptide-BSA-LA@DOX	Doxorubicin	293
			Hydrolyze peptide amide bonds of extracellular matrix proteins	Participate in tissue remodeling and metastasis	Brush peptide-polymer amphiphiles composed fluorescent nanoparticle	Fluorescence dye	294
			Hydrolyze peptide amide bonds of extracellular matrix proteins	Participate in tissue remodeling and metastasis	Micellar nanoparticles with a surface comprised of MMP-substrates and a hydrophobic paclitaxel core	Paclitaxel	292
			Hydrolyze peptide amide bonds of extracellular matrix proteins	Participate in tissue remodeling and metastasis	Phenylboronic acid conjugated human serum albumin grafted mesoporous silica nanoparticles	Doxorubicin	282
		Thrombin	Hydrolyze peptide amide bonds of fibrinogen	Participate in haemostasis, thrombosis, cell signaling, fibrinolysis and inflammation	Layer-by-layer assembly of poly(2-oxazoline)-based materials	Thrombolytic agent	308
		Thermolysin	Hydrolyze peptide amide bonds containing hydrophobic amino acids.	Produced by *Bacillus thermoproteolyticu*	Poly(L-glutamic acid) star polypeptides using PPI dendrimers as initiators.	Rhodamine B	309
		Trypsin	Hydrolyze peptide amide bonds	Pancreas	Bola-like cationic diphenylalanine nanocarriers	Doxorubicin	298
			Hydrolyze peptide amide bonds at C terminal of lysine and arginine	Produced by the pancreas, activated in the small intestine	Protamine/ sulfatocyclodextrin supramolecular nanoparticles	Trisodium salt of 8-hydroxypyrene-1,3,6-trisulfonic acid (HPTS)	299
		Proteinase K	Hydrolyze peptide bonds	Candida albicans	Methotrexate-conjugated magnetic nanoparticles and glycine coated magnetic nanoparticles	Glycine and methotrexate	296
			Hydrolyze peptide bonds	Candida albicans	Polytyrosine nanoparticles	Doxorubicin	60
	Ester bonds	Acetylcholinesterase	Hydrolyze acetylcholine and other choline esters	Present in neuromuscular junctions	Poly(ethylene glycol)-block-poly(acrylic acid) with myristoylcholine chloride	Nile red	310
		Phospholipase	Hydrolyze lipids	Present in human digestive system, intracellular compartment and extracellular spaces	(R)-1-O-hexadecyl-2-palmitoyl-snglycero-3-phosphocholine	Antitumor ether lipids	301
			Hydrolyze phosphoric acid monoester in peptide sequences	Participate in signal transduction and protein activity	ATP coated Ag nanoparticles	Silver nanoparticles	311
	Glycosidic bonds	α-amylase	Cleaved α-1,4 glycosidic bond	Present in saliva	Hydroxyethyl starch based 10-hydroxy camptothecin (10-HCPT)-HES and 5-FU-HES conjugates	Paclitaxel	312
		β- Glucuronidase	Hydrolyze complex carbohydrates	Present in lysosome, necrotic tissue, and some solid tumor types	β-glucuronidase-responsive prodrugs with the potent monomethyl auristatin E linker	Monomethyl auristatin E	313
**Oxidoreductases**	Azo compounds	Azoreductase	Reductive azo compounds	Colon bacteria	Copolymers of 2-hydroxyethyl methacrylate (HEMA) and methyl methacrylate (MMA), and terpolymers of HEMA, MMA, and methacrylic acid	Ibuprofen	291
**Transferases**	Phosphorus-containing groups	Creatine kinase	Phosphorylate hydroxyl group in peptide sequences	Regulate cellular pathways	Liposome based DSPE-PEG2000-TAT	Paclitaxel	289

**Table 8:** Clinical translation of stimuli-responsive nanocarriers

Stimulus	Nanocarriers	Cargo	Indications	Clinical status	Reference
Magnetic	Iron oxide magnetite	Iron oxide nanoparticles	Prostate cancer	Phase I	NCT02033447
	Iron and carbon (MTC-DOX)	Doxorubicin	Unresectable hepatocellular carcinoma	Phase II and III	NCT00034333
			Hepatocellular carcinoma	Phase I and II	NCT00054951
			Liver metastasis	Phase I and II	NCT00041808
Temperature	Liposomes (ThermoDox)	Doxorubicin	Recurrent regional breast cancer	Phase I and II	NCT00826085
			Liver tumor	Phase I	NCT02181075
			Pediatric refractory solid tumor	Phase I	NCT02536183
		Doxorubicin combined with high Intensity focused ultrasound (HIFU)	Painful bone metastases, breast carcinoma, non-small cell lung cancer, small cell lung cancer, adenocarcinoma	Phase II	NCT01640847
		Doxorubicin combined with standardized radiofrequency ablation	Hepatocellular carcinoma	Phase III	NCT02112656
pH	Polymeric micelles (NC6300)	Epirubicin	Solid tumor, soft tissue sarcoma, metastatic sarcoma, sarcoma	Phase I and II	NCT03168061
Secretory phospholipase A2 (sPLA2)	Liposomes (LiPlaCis)	Cisplatin	Advanced or refractory solid tumor, metastatic breast cancer, prostate cancer and skin cancer	Phase I and II	NCT01861496
